# Thermal Desorption Gas Chromatography–Mass Spectrometry Methods for Minimally Invasive Organic Residue Analysis of Archeological Potsherds

**DOI:** 10.1021/acs.analchem.5c05331

**Published:** 2025-11-13

**Authors:** Eugenia Geddes da Filicaia, Charlie A. Maule, Alexander Nixon, David A. Peggie, Ian D. Bull, Richard P. Evershed, Mélanie Roffet-Salque

**Affiliations:** † Organic Geochemistry Unit, School of Chemistry, 1980 University of Bristol, Bristol BS8 1TS, U.K.; ‡ Scientific Department, The National Gallery, Trafalgar Square, London WC2N 5DN, U.K.

## Abstract

Organic residue analysis (ORA) is widely used to investigate lipids preserved in archeological pottery. However, the amount of sample typically involved (2 g) precludes its application to particularly precious, small, or brittle sherds. This paper presents a novel, minimally invasive, protocol to overcome this limitation. By using a thermal separation probe (TSP) coupled to gas chromatography–mass spectrometry (GC-MS), this approach utilizes thermally assisted hydrolysis and methylation (THM), reducing sample sizes to as little as 1 mg. Testing with standard solutions and modern lipid-doped sherds confirmed that the method enables recovery of fatty acids (FAs) and determination of their carbon isotope compositions. The new approach was then validated using a range of archeological sherds previously analyzed using established liquid extraction-ORA approaches. In addition to fatty acids, other lipid biomarkers such as miliacin and ω-(o-alkylphenyl)­alkanoic acids (APAAs) were recovered. With the presented THM method, results comparable to established ORA methods are obtained but, crucially, sample sizes are reduced by nearly 3 orders of magnitude. This widens the application of ORA, for example, by enabling the study of valuable objects, permitting lipid profiling on different areas of one sherd, or serving as a rapid screening method ahead of compound-specific radiocarbon dating.

## Graphical abstract

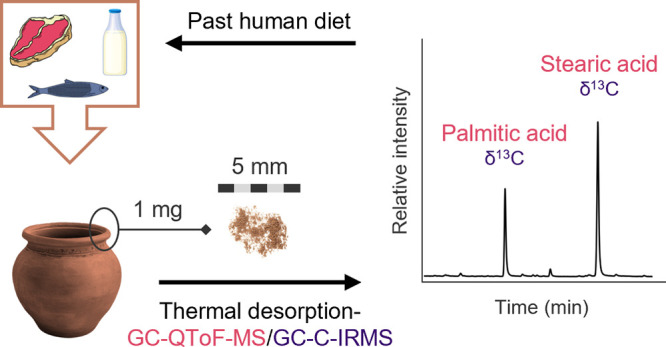

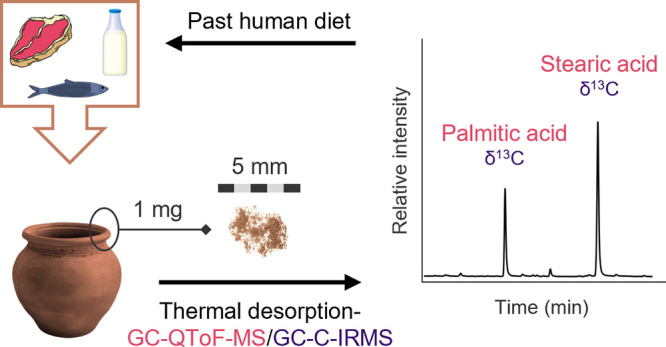

Archeological pottery vessels frequently retain associated organic remains, which may yield a wealth of information on the use of the vessel, and the economies and technologies of the site and the wider region.^1,2^ Residues absorbed within the vessel wall, hence invisible to the naked eye, are very widespread. Nonetheless, their source may often be identified through molecular characterization and determination of carbon isotope composition. These residues derive from the foodstuffs that were transported, stored, or cooked in the pottery, whose lipid components became trapped and are preserved in the pores of the clay matrix.^3,4^ In most cases, this pertains to long-chain alkyl compounds, frequently found in animal and plant waxes, as well as to triacylglycerides, main components of animal fats and plant oils serving as energy storage. Other major classes of lipids, namely sterols and phospholipids, do not survive beyond trace amounts.^5^

Compositional investigation of these residues is typically carried out with gas chromatography (GC) and mass spectrometry (MS)-based techniques, which permit characterization of a wide range of compound classes preserved in low abundance in complex mixtures of degraded organic matter. Notably, GC is utilized for the separation and quantification of simple or hydrolyzed low molecular weight lipids (e.g., fatty acids, FAs), while high temperature GC (HT-GC) for the separation and quantification of intact complex lipids such as triacylglycerides, diacylglycerides, monoacylglycerides, and wax esters. While GC-MS and HT-GC-MS are routinely used for the separation and characterization of lipids,^6^ GC-combustion-isotope ratio-MS (GC-C-IRMS) affords additional information by determining relative stable isotope compositions, most commonly δ^13^C values,^7,8^ of individual compounds.

Several methods have been developed over the years to isolate organic residues from archeological potsherds for GC analysis. The most widespread protocols utilize solvent extraction from the ground pot,^6,9,10^ most commonly with a dichloromethane/methanol (DCM/MeOH; 2:1 *v*/*v*)^11,12^ solvent system. Sonication or centrifuging facilitates the extraction of a wide variety of lipid classes deriving from animal fats, plant oils, and waxes, including FAs, long-chain ketones, wax esters, acylglycerols, *n*-alkanols, and *n*-alkanes.^3,5,6,13,14^

In some cases, this method is unable to afford complete lipid recovery, as polar lipids form strong interactions with the ceramic matrix, causing them to become insoluble in the solvent system. Treatment with a strong base, however, may recover this ‘bound’ fraction.^15,16^ To render some lipids GC-amenable, derivatization must be carried out after extraction. Most commonly, this is achieved by saponification followed by trimethylsilylation or methylation, to obtain, in the case of FAs, trimethylsilyl (TMS) or methyl ester (FAME) derivatives, respectively.^7^ Acid-catalyzed esterification, notably with boron trifluoride-MeOH complex (BF_3_-MeOH), is a common procedure to produce FAMEs,^2,17–19^ while *N,O*-bis­(trimethylsilyl)­trifluoroacetamide (BSTFA) with 1% trimethylchlorosilane (TMCS) is one of the most utilized silylation reagents.^6,15^

Spurred by the need to process large numbers of potsherds, Correa-Ascencio and Evershed (2014) developed a protocol in which lipids are extracted and transmethylated simultaneously, using an acidified MeOH solution (H_2_SO_4_/MeOH 2% *v/v*). Importantly, this method also frees the ‘bound’ fraction, yielding higher concentrations of lipids than with solvent extraction alone. Although information on intact lipids is lost as they are hydrolyzed,^20^ this has become an extremely popular protocol for ORA.^2,21–24^ Most other alternatives to solvent extraction require costly and complex equipment, including supercritical fluid extraction,^25^ the use of catalytic hydropyrolysis^16^ or a microwave-assisted recovery system,^26^ preventing fast throughput of samples.

While ORA is incredibly useful and effective for reconstructing the diets of ancient people, and technological and ritual activities, sample preparation protocols are long and complicated, requiring several days to complete. Moreover, an important limitation is that ORA, a destructive technique, requires about 2 g of pottery. This amount, suitable for archeological sites with a multitude of sherds, precludes analysis of rare or intact vessels which would be unethical to sample in this quantity.^21^ Examples include funerary pottery, such as ceramic baby bottles,^27^ or sieves (cheese-strainers).^28^ Additionally, as the protocols require the sherds to be powdered prior to solvent extraction, all spatial information on lipid distribution is lost.^29^ Recovery of lipids from smaller samples can be difficult, and increased challenges are encountered for very ancient or rare vessels. Generally, the amount of organic residue surviving decreases as the age of the object increases, with the state of preservation and amount of the organic residue also being affected by size and use of the ceramic vessel, among other things.^3,30^

GC-MS methods based on thermal degradation, most commonly pyrolysis (Py)-GC-MS, require minimal sample preparation and amounts. Although these may seem an obvious avenue to pursue to circumvent some of the limitations of established extraction methods, these types of techniques are not routinely used for ORA. This applies in particular to thermally assisted hydrolysis and methylation (THM)-GC-MS,^31^ while Py-GC-MS, without derivatization, has been utilized.^32–35^ The first study mainly investigated visible remains from potsherds, interpreted largely as food remains, but also obtained a series of normal alkane/alkene peak doublets from the analysis of the ceramic material, which the authors attributed to the heating of lipids at high temperatures during cooking.^32^ However, most other subsequent studies mainly yielded information on charred organic matter, primarily associated with the firing of the ceramic and tempering process,^33–35^ rather than on the use of the vessels during their lifetime.

Although Sanjurjo-Sánchez et al. (2018) attributed a minor proportion of recovered organic matter to food residues, no more specific information could be obtained from their data. In addition, they found THM-GC-MS with tetramethylammonium hydroxide (TMAH) to be more ineffective than Py-GC-MS. Another study, however, used THM-GC-MS to analyze Korean Neolithic potsherds, detecting palaeoculinary biomarkers, including aquatic commodities.^36^ Recently, a considerable number of prehistoric potsherds (*n* = 50) from Spain were analyzed by THM-GC-MS. The method enabled investigation of charred residues associated with the soil (or the clay or temper), and uncharred plant remains from postdepositional processes or from an unknown origin (e.g., FAs).^31^ All these studies used TMAH (25% in MeOH) as the derivatization agent, pyrolyzer temperatures between 610–750 °C, and sample weights ranging between 1–5 mg.

A variation of Py-GC-MS, recently termed direct inlet pyrolysis (DIP)-GC-MS, is achieved through the use of a thermal separation probe, or TSP. In DIP-GC-MS, low temperature Py (max 450 °C), or thermal desorption, occurs directly in the GC inlet, limiting the likelihood of losing volatile compounds during transfer to the column.^37^ Compared to conventional Py, DIP-GC-MS entails a slower temperature ramp, reducing the extent of pyrolytic cleavage.^38^ This technique has been used for the analysis of two residues from different archeological potsherds, obtaining information on the organic components from sample amounts of 20 μg or less.^39^ Interestingly, another tetraalkyl ammonium salt, 3-(trimethylfluoride)­phenyltrimethylammonium hydroxide (TMTFTH), able to achieve THM at the normal operating temperatures of GC injectors (a minimum of 220–290 °C^40^) was used in another study on ORA of Late Bronze Age Egyptian amphorae. The results showed that lipids could be extracted with this method, proving more effective than solvent extraction. Moreover, sample sizes utilized (0.1 g) were considerably lower than in typical protocols.^41^

Through the development of new analytical techniques, this paper proposes a novel minimally invasive protocol for ORA of archeological sherds. By lowering the required sample size from 1 to 2 g to as little as 1–3 mg, the new method broadens the application of this type of analysis. This is achieved by coupling a TSP to a gas chromatograph-quadrupole time-of-flight-mass spectrometer (GC-QToF-MS) and, separately, a GC-C-IRMS, enabling thermal desorption and THM of archeological lipids directly in the GC inlet. The new techniques are termed DIP-GC-QToF-MS and DIP-GC-C-IRMS.

## Materials and Methods

### Standards

Standard solutions (0.005 mg mL^–1^ for GC-QToF-MS, 0.1 mg mL^–1^ for other instruments) were prepared by dissolving lipids in DCM. These included a saturated FA solution, composed of C_14:0_, C_15:0_, C_16:0_, C_17:0_, C_18:0_, and C_20:0_ FAs, an unsaturated FA (UFA) solution, containing C_18:1_ and C_22:1_, and a triacylglycerol (TAG) solution, comprising TAGs C_14:0 × 3_, C_16:0 × 3_, C_18:0 × 3_, and C_18:1 × 3_. A solution containing C_21:0_ FA (FA_21:0_) in DCM/MeOH (2:1 *v/v*), serving as internal standard (IS) for quantification purposes, was prepared at the same concentrations.

### Modern Doped Sherds

A modern clay pot was broken in pieces of about 1 g each, and furnaced (450 °C, 2 h) to ensure complete removal of organic matter. These were then doped with FA, UFA, and TAG standard solutions (1 mg mL^–1^) to achieve different concentrations of lipids (10, 25, and 50 μg g^–1^ per standard solution). The solution was added to the sherd gradually, by microsyringe, followed by DCM, to increase the likelihood of homogeneous dispersal throughout the sherd. The total amount of liquid used was 100 μL per sherd. After evaporation of the DCM (2 h) the sherds were crushed to powder with a mortar and pestle.

### Preparation of Archeological Potsherds and Lipid Extraction and Derivatization with Established Protocols

Eight potsherds (6 g each) from three different sites (1, 2, and 3) were cleaned with a rotary tool and crushed (see Table 1 for relevant characteristics for their choice in this study, identified during previous analysis). The powder was then divided into 3 portions (2 g each). Following published protocols,^6,12,20,42,43^ one portion was solvent extracted (DCM/MeOH, 2:1 *v/v*) and derivatized with BSTFA, while another portion was extracted and derivatized with acidified MeOH (4% H_2_SO_4_). The last portion was kept for THM.

**Table 1:** Potsherds Analyzed, With Relevant Characteristics

Sherd	Conc. of lipids	δ^13^C value	Biomarkers	Site number
LDW2235	Low	Medium	–	1
LDW2263	Low	Medium	–	1
TUR0234	Medium	High	Miliacin	2
TUR0244	Medium	High	Miliacin	2
MYL0766	High	Low	Aquatic^44^	3
MYL0873	High	Low	Aquatic	3

### Thermally Assisted Hydrolysis and Methylation (THM)

Standard solution (1 μL), or powdered sherd (1 mg for DIP-GC-QToF-MS, 1–3 mg for DIP-GC-C-IRMS) was placed in a furnaced (450 °C, 1 min) TSP tube. The IS was added (1 μL) together with trimethylsulfonium hydroxide (TMSH), 2 μL, 0.02 M for DIP-GC-QToF-MS and 0.25 M for DIP-GC-C-IRMS. The solvent was allowed to evaporate at room temperature before analysis. Each sample was analyzed in triplicate (DIP-GC-QToF-MS) or duplicate (DIP-GC-C-IRMS).

### Instrumentation

TMS derivatives were screened and quantified using HT-GC-FID, and identified through HT-GC-QToF-MS. FAMEs resulting from the acidified MeOH protocol were screened and quantified using GC-FID and identified through GC-MS, while aquatic biomarkers were identified by GC-QToF-MS. FA δ^13^C values of FAMEs were determined through GC-C-IRMS. FAMEs resulting from the THM protocol were quantified and identified using DIP-GC-QToF-MS. FA δ^13^C values were determined through DIP-GC-C-IRMS. See S1 for details on instrument methods.

## Results and Discussion

### Recovery of Lipids from Standard Solutions

The FA, UFA, and TAG standard solutions were analyzed at 0.005, 0.0075, and 0.01 mg mL^–1^ with DIP-GC-QToF-MS. Results show that TMSH is able to methylate saturated and unsaturated FAs to yield the corresponding FAMEs, and to transesterify TAGs to FAMEs, following previously published mechanisms.^45^ The recovery of FAs was much lower for UFAs and TAGs than for the free saturated FAs. Figure 1 shows the recovered FA amounts, calculated using FA/IS peak area ratios, for each triplicate analysis (A, B, and C, and their average) for the FA standard solutions (see Figure S1 for UFA and TAG standard solutions, and Tables S1–S3 for amounts of recovered FAs). For the FA and UFA standard solutions, the theoretical amount expected (marked Theor., in yellow on the bar chart) for each corresponding FAME is 5, 7.5, and 10 ng per analysis, respectively, for the 0.005, 0.0075, and 0.01 mg mL^–1^ solutions. For FA solutions, the average calculated FAME amounts are consistent with the theoretical amounts for all three concentrations, with a maximum deviation of ±2.5 ng. The standard deviation (SD) between triplicate analyses generally falls between ±1 to ±3 ng, indicating good reproducibility, with a maximum of ±6 ng (only on one occasion).

**Figure 1:**
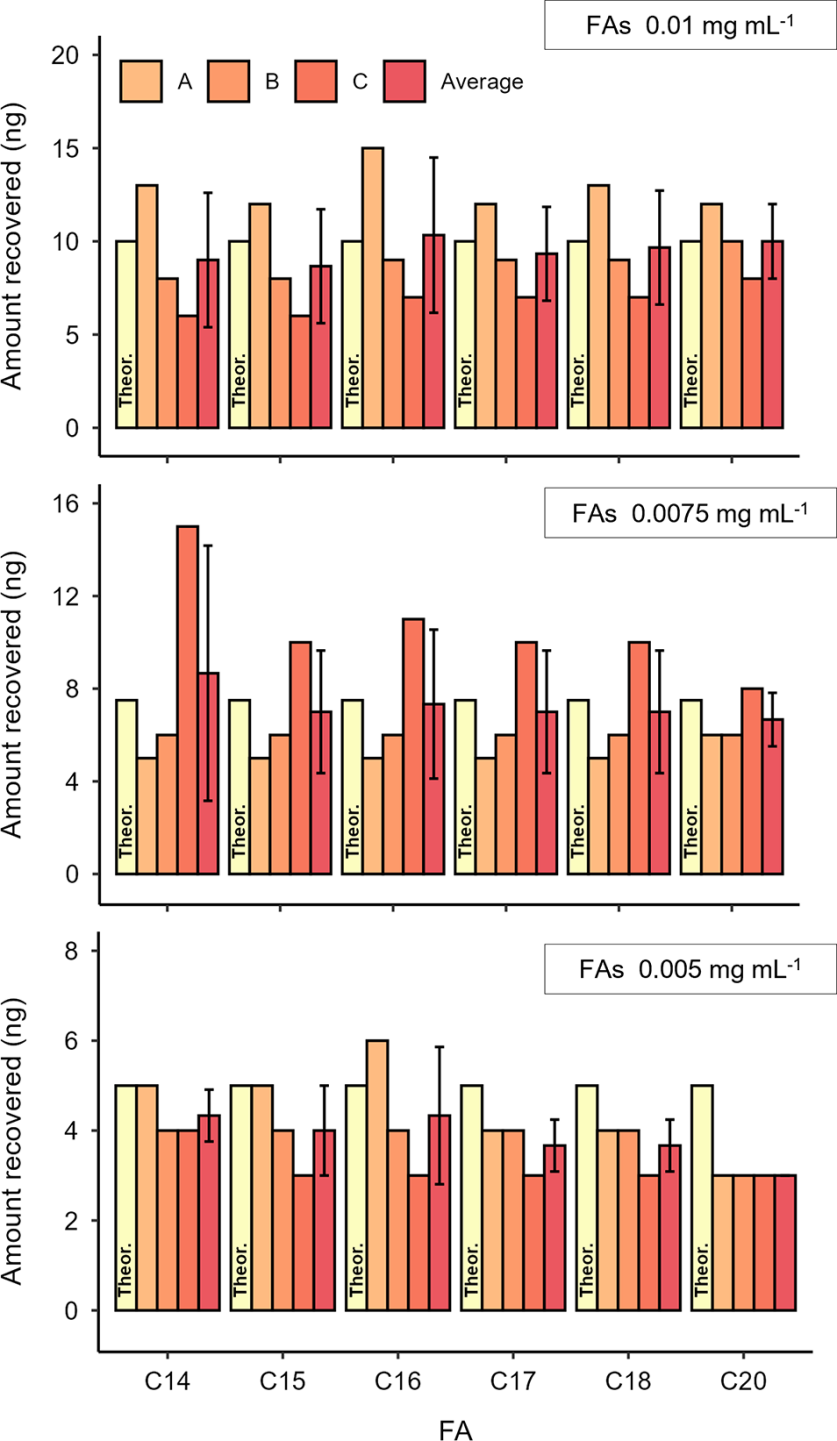
Amounts of FAs recovered from THM by DIP-GC-QToF-MS, performed in triplicate (A, B, C), of FA standard solutions (1 μL). The average value is reported with the SD. Theoretical (Theor.) amount expected in yellow.

Calculated amounts of recovered FAs from analysis of the UFA standard solutions ranged between 1 and 2 ng for each C_18:1_ and C_22:1_ for the 0.0075 and 0.01 mg mL^–1^ solutions, and between 0 and 1 ng for the 0.005 mg mL^–1^ solution. The amount of FAs recovered from the TAGs standard solutions, in case of complete transesterification, was expected to be 15, 22.5, and 30 ng for each FA, respectively, for the 0.005, 0.0075, and 0.01 mg mL^–1^ solutions. Yields, however, ranged between 0 and 1 ng per FA for the lowest concentrations, and between 0 and 6 ng (with SD between ±1 and ±3 ng for each triplicate) for the 0.01 mg mL^–1^ solution.

### Recovery of Lipids from Doped Modern Sherds

Analysis of modern sherds doped with the above FA, UFA, and TAG standard solutions (at 10, 25, and 50 μg g^–1^) confirm that, through thermal desorption, it is possible to recover lipids from as little as 1 mg of powdered sherd. Figure 2 shows the typical GC-MS total ion chromatogram (TIC) obtained from the FA-doped sherds (see Figure S2 for corresponding examples for UFA- and TAG-doped sherds). The lipid profiles, together with the rates of recovery, reflect those observed from the corresponding standard solutions. It should be noted that, in this case, larger variations between lipid recovery of triplicate measurements were anticipated, as lipid deposition during doping is not expected to have been homogeneous. Thus, SD should only be used as a rough guide to assess repeatability.

**Figure 2:**
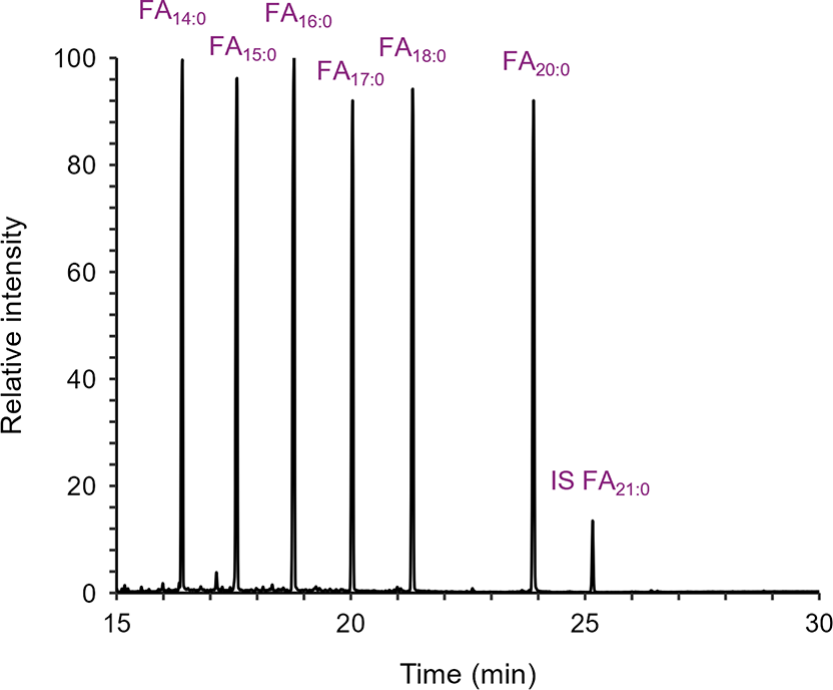
GC-MS partial TIC showing FAs recovered from THM by DIP-GC-QToF-MS of crushed modern sherd doped with FA standard solution (50 μg g^–1^).

Assuming homogeneous deposition, theoretical amounts for each FA recovered from the saturated FA- and UFA-doped sherds are expected to be 11, 25, and 50 ng per triplicate, respectively, for the 10, 25, and 50 μg g^–1^ doped sherds. For TAG-doped sherds, in case of complete transesterification, theoretical recovered amounts would equal 30, 68, and 138 ng for each concentration, respectively. The amounts of FAs recovered from all doped sherds are summarized in Figure S3, and listed in Tables S4–S6. For saturated FA-doped sherds, these show a high and consistent recovery of all FAs, proportional to increasing lipid concentration. Calculated amounts are generally at the same order of magnitude within triplicate measurements, with only one analysis (C, 50 μg g^–1^ doped sherd) exhibiting substantial differences. For UFA- and TAG-doped sherds, recovered FA amounts are generally 20% or less of the theoretical value for UFAs, and 33% or less for TAGs. For the latter, recovery of C_14:0_ and C_16:0_ FAs is higher than C_18:1_ and C_18:0_ FAs, except for sherds with the highest lipid concentration (50 μg g^–1^). This suggests that, at low levels, saturated TAGs are more efficiently transesterified by TMSH than monounsaturated TAGs. In addition, saturated TAGs of lower molecular weight, C_14:0 × 3_ and C_16:0 × 3_, are more efficiently derivatized than the heavier C_18:0 × 3_.

### Assessment of Lipid Recovery through THM by DIP-GC-QToF-MS

TMSH was chosen as a derivatizing agent due to its mild alkalinity and high efficiency of derivatization for PUFAs,^46–48^ which have been shown to isomerize or degrade when analyzed with TMAH. In contrast to the results described above, Evans et al. (2003) reported a high yield of unsaturated FAMEs, comparable to that obtained for saturated FAMEs, from THM of FA standards with TMSH. These inconsistencies, however, may be explained by the different reaction conditions of the systems used. Indeed, the efficiency of THM with TMSH for UFAs and PUFAs has been shown to vary wildly, depending on a variety of factors such as temperature of the reaction, type of injection system, concentration of reagent,^48^ reagent/analyte ratio, and time of interaction of the two. In the present study TMSH (0.02M) was diluted by a factor of 10 from the typical concentration (0.25M), to avoid chemical contamination. The previously reported phenomenon of stationary phase degradation by reaction with TMSH^49^ is believed to be particularly problematic with this system, due to the low concentrations of the target analytes and high detection limits of the instrument.

Furthermore, the amounts of each FA analyzed herein (as little as 0.005 μg) were significantly lower than those in previous studies (e.g., 1 μg; ref 45). Although TMSH was still present in excess, it is likely that at these concentrations analyte and reagent molecules had lower opportunity to interact in the relatively large volume of the inlet liner, especially considering the slower temperature ramp of the DIP-GC-QToF-MS system compared to flash pyrolysis in Py-GC-MS. This is supported by another study^50^ that investigated THM reactions of saturated FA, UFA, and TAG standards with different reagents, pyrolysis temperatures and injection systems. Although this study did not include a multimode inlet (MMI, required for a TSP), they observed that recovery of unsaturated FAMEs was less than 15%, and generally lower than that of saturated FAMEs, when using a PTV injector with a temperature ramp starting at 60 °C and reaching 200–400 °C (heating rate of 720 °C min^–1^). When using pyrolysis, a higher recovery of unsaturated FAMEs was obtained. Similarly, complete hydrolysis and methylation of TAGs to FAMEs was not obtained with any reagent with PTV injection, and complete transmethylation of TAGs containing UFAs was not achieved in any case.

UFAs, whether free or as TAG-bound moieties, were characterized by the lowest yield compared to their saturated counterparts. In the case of the UFA standard solution, it is believed that this is also partly due to a degree of evaporation of the compounds from the TSP tube before analysis. This factor is difficult to control, as the tubes are manually loaded with a high amount of solvent (4 μL) which has to evaporate before insertion in the TSP. The different conditions in the laboratory (ambient temperature and humidity) will also affect the rate of evaporation, especially in the summer, when temperatures (in the UK) may reach just below 30 °C. It was observed that, with 2 days separation between loading of the sample in the TSP tubes and analysis, FAME peaks for the C_18:1_ and C_22:1_ FAs were absent from the GC-MS TIC. This was not observed for the saturated FAs. Hence, in all analyses discussed herein, the amount of time elapsing between injection of the standards in the TSP tubes and analysis was minimized to about 30 min.

## Comparison of Archeological Lipid Profiles Obtained with Different Protocols

### Solvent Extraction and Trimethylsilylation

The GC-MS TICs obtained from all archeological sherds yielded lipid distributions typical of degraded animal fats.^2,6,51^ As shown in the example (sherd TUR0234) in Figure 3a, profiles were dominated by the presence of TMS esters of FA_16:0_ and FA_18:0_, and lower amounts of FA_14:0_ and FA_20:0_. Odd-numbered and branched-chain FAs (C_15:0_, C_17:0_, C_17:0br_, and C_19:0_), characteristic of ruminant fats and identified as biomarkers for rumen bacterial population,^51,52^ were detected in all sherds. FA_18:1_, present in high amounts in fresh animal fats, was found in low amounts in all extracts, as expected from archeological lipids having undergone oxidation during vessel use and burial.^51^ However, the two sherds from Site 2 exhibited higher amounts of FA_18:1_ which, along with the detection of a variety of mono- and diacylglycerols (MAGs and DAGs, respectively), not detected in the other extracts, highlights the higher degree of lipid preservation in these sherds. Furthermore, the detection of midchain ketones (Ks) with carbon numbers *n*-C_31_ and *n*-C_33_ (M^+·^ 450 and 478, respectively, with common base peak *m*/*z* 239) suggests that the animal lipids in these sherds have been subjected to high temperatures, probably during cooking.^53^ The presence of the short-chain FA *n*-C_9:0_ in most of the chromatograms can be attributed to the degradation of oleic acid, which may cause scission of double-bonds resulting in free monocarboxylic acids.^54^

**Figure 3:**
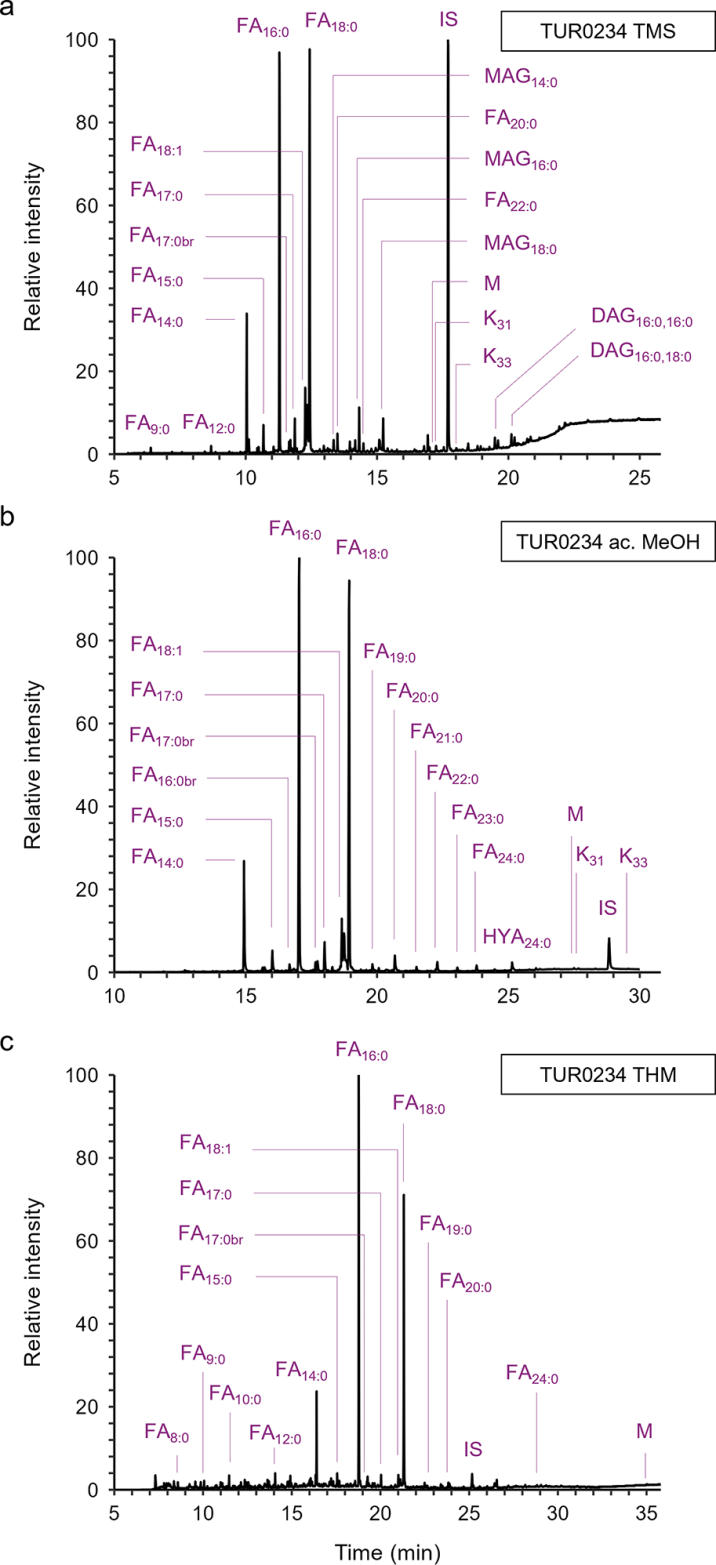
GC-MS partial TIC showing lipids recovered from sherd TUR0234, analyzed by solvent extraction (a), acidified MeOH (b), and THM (c). DAG = diacylglycerol, FA = fatty acid, K = midchain ketone, HYA = hydroxy acid, M = miliacin, MAG = monoacylglycerol.

### Direct Hydrolysis and Methylation

Analysis of sherd extracts yielded similar lipid distributions as above, with GC-MS TICs dominated by C_16:0_ and C_18:0_ FAMEs with lower abundance of C_14:0_ and C_20:0_ FAMEs (see Figure 3b for TUR0234). However, as expected, no TAGs or DAGs are present in this case, having been fully hydrolyzed and methylated to their corresponding FAMEs. Very low amounts of MAGs were detected in the extracts from sherds LDW2454 and LDW2472. In contrast to the solvent extracts discussed above, longer-chain FAMEs, typically C_22:0_ but also C_23:0_, C_24:0_, and C_26:0_ are observed for most sherds in low abundance. In addition, various α-hydroxy FAs (HYAs, present as TMS derivatives), namely C_17:0_ (sherd LDW2235), C_18:0_ (LDW2235, LDW2263, MYL0766), C_23:0_, and C_24:0_ (in the sherds from Site 2) were detected in some of the extracts. These type of compounds, produced from oxidation of FAs,^51^ have been shown to be preserved in pottery through chemical bonding with the inorganic matrix, and may be released with this extraction method^20^ (in contrast to solvent extraction protocols).

### THM with TMSH

Similarly to the acidified MeOH protocol, GC-MS TICs are characterized by the presence of FAMEs of different carbon chain lengths, with C_16:0_ and C_18:0_ predominating (see Figure 3c for TUR0234). The baseline is generally noisier, especially when the concentration of lipids in the sherd is low. This is attributed mainly to the presence of TMSH, its thermal degradation products when present in excess, and the fact that it is known to react, to some extent, with the stationary phase of the capillary column.^49^ No hydroxy FAMEs were detected in the chromatograms, but C_19:0_ and C_20:0_, and less frequently C_22:0_, C_23:0_, and C_24:0_ FAMEs, were present in low abundance. In addition, shorter chain FAMEs, namely C_8:0_, C_9:0_, C_10:0_ and C_12:0_, not frequently detected by the other methods, were present in the TICs. All other FAMEs detected with both the acidified MeOH and solvent extraction protocols, C_14:0_, C_15:0_, C_17:0br_, and C_18:1_, were also detected, in similar ratios, with THM. The midchain ketones *n*-C_31_ and *n*-C_33_, identified in TUR0234 and TUR0244 with the other methods, were not found in this case. Possibly, under the analysis conditions used, these compounds failed to be desorbed from the clay matrix, or, alternatively, they react with TMSH.

Some contaminant peaks were ubiquitously present, such as phthalates, adipates, and benzoic acid alkyl esters. These are common laboratory contaminants which, given the low analyte concentrations detectable using the QToF, are particularly noticeable with this technique. In addition, as the method requires transporting and weighing of samples in TSP tubes as well as manual insertion and retraction of the probe from the inlet, some low level contamination is unavoidable. Other compounds, including phenanthrene and series of normal alkenes and alkanes, were always present in THM analyses. These are attributed to thermal reactions occurring in the inlet, more obvious at low analyte concentrations.

Lastly, a modern furnaced sherd was analyzed in triplicate to function as a blank. Among TMSH-related chemical contamination products, minor levels of C_16:0_ FAME were present (1.2 μg g^–1^ ±0.7). In two out of three analyses, C_14:0_ FAME was also identified, albeit in lower amounts (0.4 μg g^–1^ ±0.2), while C_18:0_ FAME was only present in one out of three analyses (0.2 μg g^–1^). Results suggest that low levels of these FAMEs, C_16:0_ in particular, may be present due to laboratory contamination, and call for caution in the interpretation of amounts around 1 μg g^–1^ of recovered lipids. GC-MS TICs obtained for all other archeological sherds with the three methods of analysis are displayed in Figures S4–S10.

### Comparison of Archeological Lipid Recovery Amounts Obtained with Different Protocols

As discussed above, variation in lipid recovery between triplicate measurements with THM was expected, especially as the amount of sherd analyzed was substantially lower than with standard protocols. Despite this, for the same sherd, the calculated concentrations were in the same order of magnitude between triplicate analyses, with only LDW2472 displaying substantial differences (B presents a lipid concentration over four times higher than C). The amounts of FAs recovered from triplicate analyses of all archeological sherds are plotted in Figure S11 and listed in Table S7. More importantly, as displayed by the bar chart in Figure 4, the calculated average lipid mass fractions (in orange) are higher than those obtained from the solvent extraction protocol (in pink) in five out of the eight sherds. Four of these five sherds are the ones categorized in the ‘low’ and ‘medium’ lipid mass fraction range by previous analyses. Possibly, this indicates that the THM method performs better than traditional protocols in extracting lipids from lipid-poor sherds. For the other three sherds the average calculated lipid concentrations, albeit slightly lower, are in the same order of magnitude as those obtained through solvent extraction.

**Figure 4:**
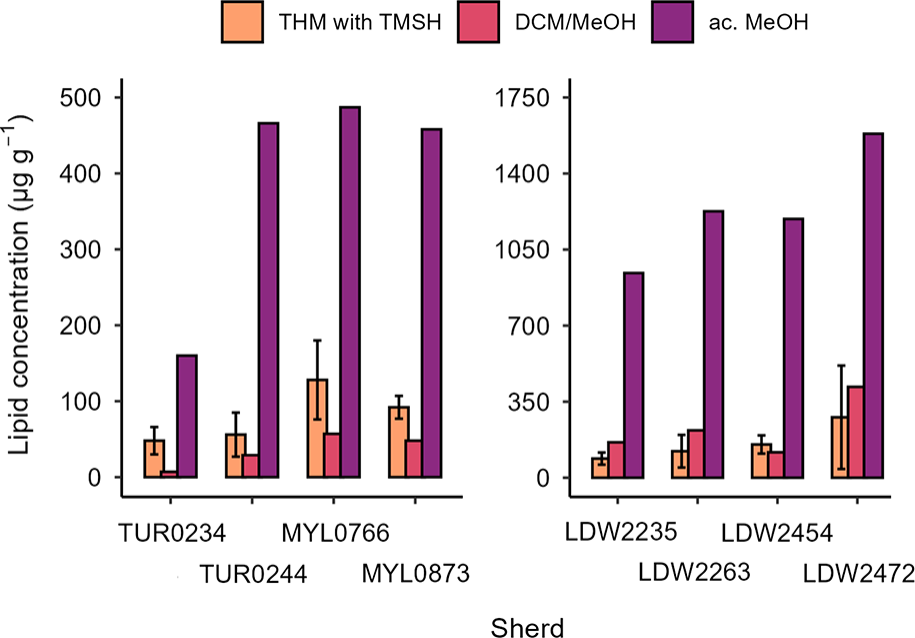
Archeological sherd lipid concentration, calculated from lipids retrieved by THM (orange, average with SD), solvent extraction (pink), and acidified MeOH (purple) protocols.

The acidified MeOH protocol (in purple in Figure 4), as expected, afforded a much higher yield of lipids than both the solvent extraction and THM methods. Specifically, the increase in yield compared to solvent extraction was between 96% and 74% (87% on average), and between 91% and 70% (83% on average) compared to THM. These results confirm that the newly developed THM method does not release the ‘bound’ lipid fraction from the ceramic matrix, and suggests that the recovered FA fraction mainly consists of free FAs and a proportion of hydrolyzed TAGs. Nonetheless, the performance of the THM method is comparable to traditional solvent extraction in the concentration of lipids it can recover. Lipid concentrations plotted in Figure 4 are listed in Table S7.

### Presence of a Cereal Biomarker, Miliacin

This cereal biomarker, when present in archeological vessels, usually survives in low amounts, and is thus best detected using GC-MS with SIM acquisition. Recently, miliacin has been unequivocally identified in archeological potsherds from Site 2, including in TUR0244.^55^ Thus, in the present study, GC-MS TICs obtained from both sherds from this site were inspected for the presence of miliacin by searching either for ions *m*/*z* 189 (base peak) and 440 (M^+·^, for acidified MeOH by GC-MS quadrupole) or *m*/*z* 189.1611 and 440.4160 (for solvent extraction and THM by GC-QToF-MS). The cereal biomarker was detected, for both sherds, in all analyses including, as illustrated in Figure 5, those resulting from the newly developed THM-based protocol. This confirms that THM is comparable with standard methods even for the detection of biomarkers at trace levels, and expands the range of compound classes (miliacin is a triterpenoid) identifiable by this method.

**Figure 5:**
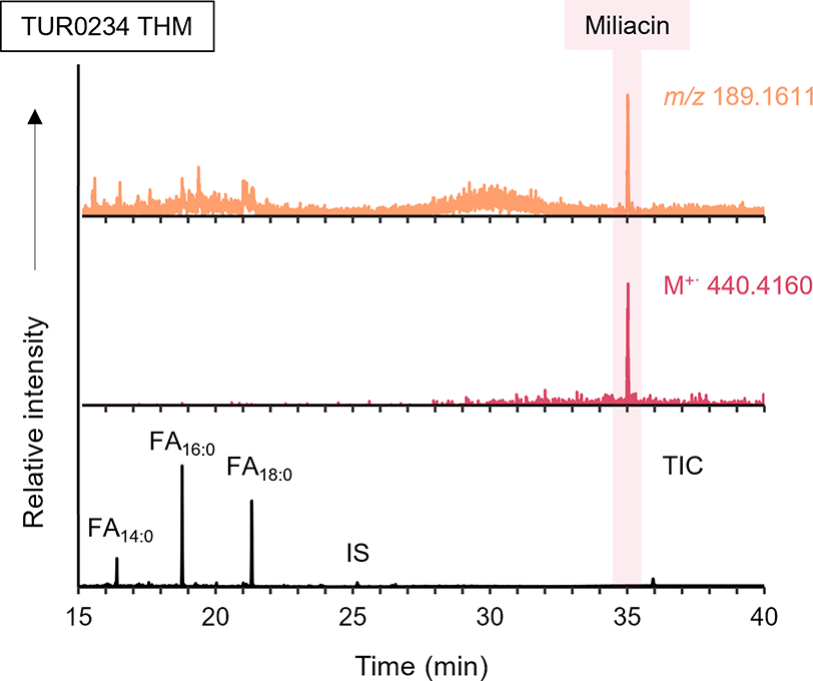
Partial TIC from DIP-GC-QToF-MS of sherd TUR0234, and the EICs for the base peak (*m*/*z* 189.1611, orange) and the molecular ion (M^+·^ 440.4160, pink) of miliacin.

### Presence of Aquatic Biomarkers

Aquatic biomarkers, if present in archeological pottery, are usually found at very low levels. Through GC-MS analysis of their extracts in SIM mode, they were detected in sherds MYL0766 and MYL0873. The presence of ω-(*o*-alkylphenyl)­alkanoic acids (APAAs) was confirmed through the extracted ion chromatograms (EICs) of molecular ions for C_18_ (M^+·^ 290), C_20_ (M^+·^ 318), and C_22_ (M^+·^ 346) APAAs (Figure S12), along with the common base peak (*m*/*z* 105).^44,56^ In the present study, in addition to the THM protocol, the acidified MeOH extracts of the sherds were analyzed by GC-QToF-MS (‘aquatic biomarker method’^57^) thus affording another way to assess the results achievable through DIP-GC-QToF-MS. In both cases, as shown in Figure 6 for sherd MYL0873, C_18_ and C_20_ APAAs were identified in the TICs, as well as all three isoprenoid acids (IPAs) shown to be important to recognize the processing of aquatic lipids in archeological pottery. As expected, they are present in low amounts, and were detected by searching for specific fragment ions of known accurate mass, namely the base peak (B^+^) and molecular (M^+·^) ions (see Table S8). Similar results were obtained for MYL0766.

**Figure 6:**
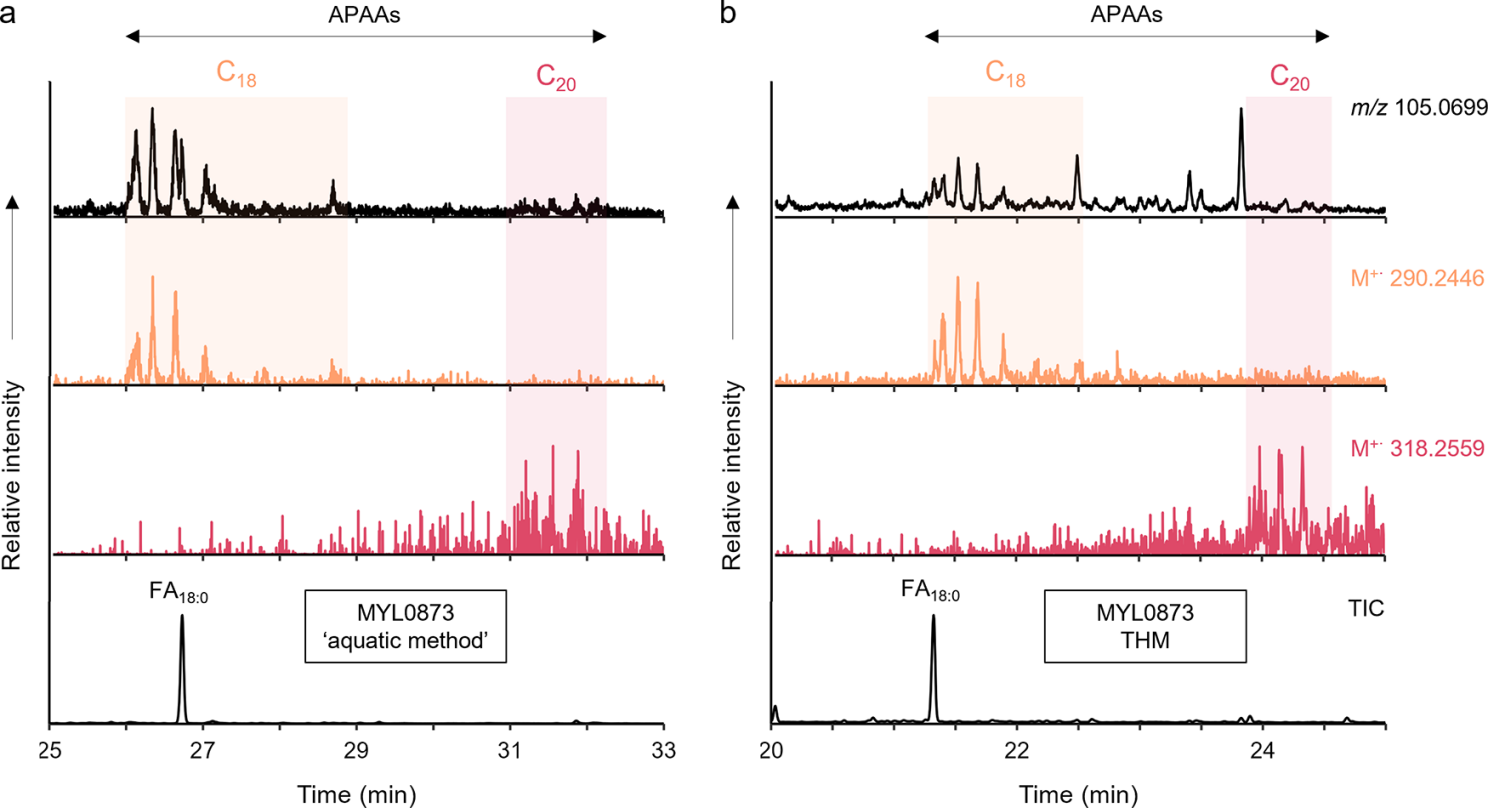
GC-MS partial TIC obtained from (a) the acidified MeOH protocol and (b) THM of sherd MYL0873. Above, the EICs for the base peak (*m*/*z* 105.0699, black) and the molecular ions of APAAs C_18_ (M^+·^ 290.2446, orange) and C_20_ (M^+·^ 318.2559, pink).

### DIP-GC-C-IRMS To Obtain FA δ^13^C Values in Archeological Potsherds

#### Validation of THM Using FA Standards

Py-GC-C-IRMS is not typically used to investigate archeological pottery. However, early work on analytical pyrolysis in the 1990s showed that there are no substantial fractionations between precursors and pyrolysates. Hence, Py-GC-C-IRMS can be reliably used for compound-specific isotope analysis,^58–60^ as demonstrated by a variety of studies on natural and synthetic polymers,^61,62^ plant carbohydrates,^60,63^ speleothems,^64^ meteorites,^65,66^ sediments and soils^45,58,67–74^ and aquatic humic substances.^75–77^ Nonetheless, in the present study, a TSP was coupled to a GC-C-IRMS for the first time. It was thus essential to validate the derivatization procedure, ensuring that any associated fractionation effects are reproducible, and, if necessary, corrected for.

The δ^13^C value of the underivatised FA (δ_c_) may be obtained by correcting the FAME δ^13^C value resulting from DIP-GC-C-IRMS (δ_cd_), if the δ^13^C value of the derivative group (δ_d_) is known, by rearranging the mass balance equation:^78^

$$n_{cd}\delta_{cd}=n_{c}\delta_{c}+n_{d}\delta_{d}$$

where *n* relates to the number of carbon atoms, *c* the target compound, *cd* the derivatized compound, and *d* the derivative group. However, if reproducible kinetic isotope effects (KIEs) are present, these must be accounted for in the above equation by substituting a correction factor (δ_corr_), which incorporates the KIEs and the δ^13^C value of the derivative group, for δ_d_.

Fractionations occur due to KIEs, arising from differences in rates of reaction or transport between isotopic species. The most important KIE is the primary isotope effect, resulting from a bond modification in a specific position during the rate-determining step.^45^ In derivatization reactions these effects are expected to occur when a bond involving the derivative carbon atom is broken and when reaction with that atom is nonquantitative. Indeed, KIEs have been shown to arise in a wide range of organic compounds analyzed by GC-C-IRMS,^78^ including FAs analyzed by THM with Py-GC-C-IRMS. In this case, the KIE was found to be robust and reproducible for each FA, so that a correction value could be used to determine their δ^13^C values.^45^

The same procedure was followed in this study, where a standard solution containing FAs (of known δ^13^C values) was analyzed by DIP-GC-C-IRMS to obtain δ^13^C values of FAMEs (measured δ_cd_). These were found to be between 1.5–2.2‰ lower than predicted δ_cd_ values, confirming the occurrence of a KIE. The predicted δ_cd_ values were obtained by rearranging the mass balance equation to solve for δ_cd_, and using EA-IRMS analysis of TMSH to obtain a known value for δ_d_. The KIE for each FA was calculated^45^ and found to be robust and reproducible, confirming that δ_corr_ could be used to determine δ_c_ values. The mean KIE, with a value of 1.033 (SD = 0.006), was similar to the analogous reaction by Py-GC-C-IRMS.^45^ The measured and predicted δ_cd_ values, and calculated KIEs and δ_corr_ values, are listed in Table 2.

**Table 2:** KIE and δ_corr_ Values for THM by DIP-GC-QToF-MS of 6 FAs, with Parameters Used for Calculations

FA	δ_c_ (‰)^a^	Meas. δ_cd_ (‰)^b^	Pred. δ_cd_ (‰)^c^	Δ (‰)^d^	KIE^e^	δ_corr_ (‰)
C14:0	–26.9	–30.7 (±0.1)	–28.3	2.3	1.035	–83.6 (±0.1)
C15:0	–31.6	–34.2 (±0.1)	–32.6	1.6	1.025	–73.9 (±0.1)
C16:0	–29.2	–32.6 (±0.1)	–30.3	2.2	1.038	–86.2 (±0.2)
C17:0	–30.7	–33.2 (±0.2)	–31.7	1.5	1.027	–75.1 (±0.2)
C18:0	–28.8	–32.0 (±0.1)	–29.8	2.1	1.040	–88.9 (±0.1)
C20:0	–28.1	–30.7 (±0.1)	–29.1	1.7	1.035	–83.1 (±0.1)

a Measured by EA-IRMS.

b Values are means (*n* = 6).

c Calculated by rearranging the mass balance equation, where δ_d_ = −48.5‰.

d Δ = meas. δ_cd_ – pred. δ_cd_.

e Calculated as in Evans et al. (2003).

#### Assessment of Chromatography and Required Sample Amounts

DIP-GC-C-IRMS of the doped modern sherds confirmed that it is possible to thermally desorb FAs with this technique, using an equivalent amount of sample as for DIP-GC-QToF-MS. Regarding the archeological sherds, different amounts of sample (1–3 mg) were analyzed, depending on the lipid concentration previously calculated. Suitable TICs were obtained for all sherds, even for sample weights as low as 1 mg. Importantly, it was noted that contact of the powdered sherd with the derivatization agent was fundamental for a good yield. For example, by increasing the amount of TMSH added (from 1.5 to 3 μL) and ensuring wetting of the whole sample for the analysis of TUR0244, the intensity of FAME_16:0_ and FAME_18:0_ peaks dramatically increased (see Figure S13).

#### Comparison of Results between Acidified MeOH Extraction and THM GC-C-IRMS Analysis

δ^13^C values for C_16:0_ and C_18:0_ FAMEs were successfully determined for all archeological sherds analyzed by DIP-GC-C-IRMS. For comparison, the corresponding acidified MeOH extracts were analyzed by the same GC-C-IRMS instrument in liquid injection mode. In this case, δ_d_ (rather than δ_corr_) was utilized in the mass balance equation (δ_d_ = δ_MeOH_ = −42.8‰, determined by EA-IRMS) as no KIE occurs with this derivatization.^78^ The δ^13^C values for C_16:0_ and C_18:0_ FAMEs obtained by both methods are analogous, while the corresponding Δ^13^C values (Δ^13^C = δ^13^C_18:0_ – δ^13^C_16:0_) are nearly identical (these are listed in Table S9, and plotted in Figure S14). The Δ^13^C proxy, as explained elsewhere,^1^ may differentiate between ruminant adipose, ruminant dairy, and nonruminant adipose animal fats. Thus, analogous interpretations are drawn from determinations performed by both methods. An in-depth analysis of the individual sherds is omitted, as it is beyond the scope of this study. Furthermore, given the small numbers of sherds analyzed, it would not serve to give an overview of archeological activities of each site.^12^

The similarity between Δ^13^C values calculated with both methods is illustrated in Figure 7, which displays the Δ^13^C value for each sherd obtained through DIP-GC-C-IRMS (orange squares) and GC-C-IRMS (pink circles). It should be noted that some variation in the δ^13^C values obtained from each method is expected, as the absorbed lipid residue is an integrated signal derived from the many uses of the vessel throughout its lifetime. Hence, the two methods may recover lipid fractions that may reflect different periods of the life/use of the vessel.^20^ The acidified MeOH protocol is known to release bound lipids from ceramic vessels, while the new THM protocol does not. This might contribute to the slight variation in values, although the absence of a ‘bound’ lipid fraction has been shown to not appreciably alter data interpretation.^16^

**Figure 7:**
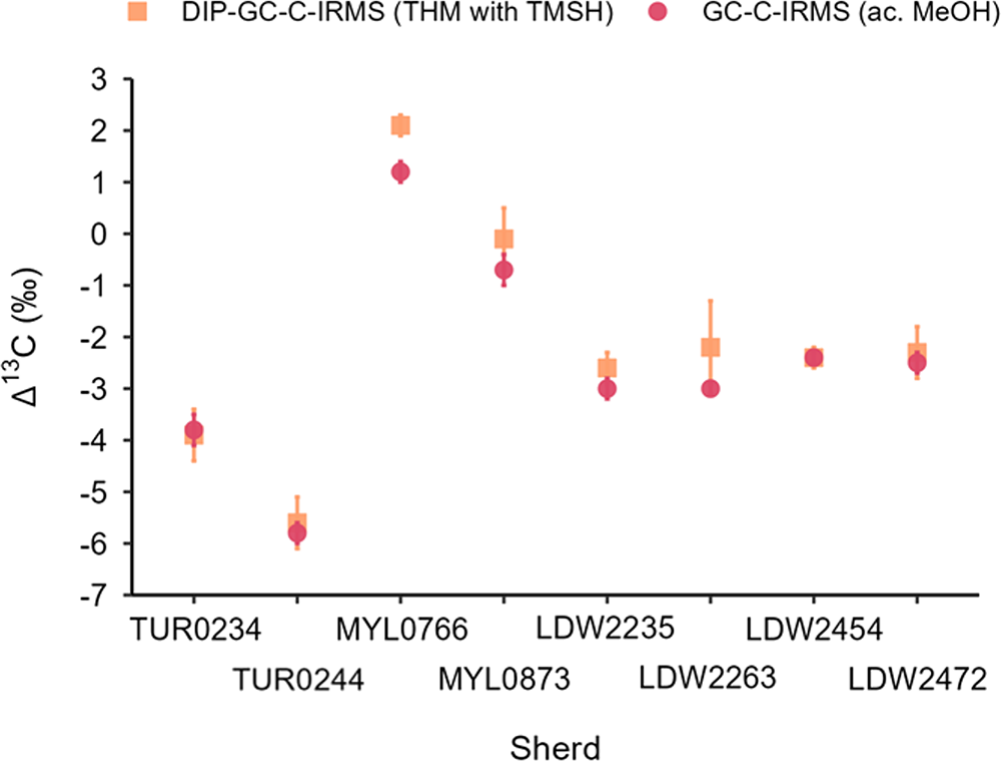
Δ^13^C values (‰) for archeological sherds obtained by THM (orange squares) and acidified MeOH protocols (pink circles). The error bars denote the SD on either side (±) of the value (*n* = 2).

Generally, the error associated with the δ^13^C values determined by DIP-GC-C-IRMS (ranging between ±0.1‰ and ±0.4‰) is slightly lower than that obtained by GC-C-IRMS (ranging between ±0.1‰ and ±0.5‰). A larger error (±0.9‰) was only obtained for the δ^13^C_16:0_ value of sherd LDW2263. This is possibly due to the low concentration of lipids in the portion of the sherd used for the second analysis, which exhibited FAME peaks (in particular the C_16:0_ peak) of the lowest intensity among the sherds investigated.

## Conclusions

ORA is an established field of study for investigating the type and source of lipids preserved in archeological pottery. Nonetheless, the amount of sample typically involved (2 g) precludes its application to archeological sites which do not display a high abundance of sherds, for vessels which are particularly valuable, exceptionally brittle, or degraded. To overcome this limitation, this paper describes a novel, minimally invasive, ORA approach based on thermal desorption. The study describes how, by means of a TSP, THM with TMSH by DIP-GC-QToF-MS and DIP-GC-C-IRMS may be used to undertake fatty acid and lipid biomarker analysis on archeological potsherds in a comparable manner to established protocols. Although the lipid ‘bound’ fraction is not extracted, the new THM method, crucially, requires as little as 1 mg for each analysis against the 2 g typically utilized by solvent extraction protocols. Possible limitations arising from inhomogeneous lipid distribution may thus be mitigated by undertaking multiple analyses.

The new THM method was first tested with lipid standard solutions of known concentrations, and on modern furnaced sherds doped with these solutions. Analysis confirmed that the method is able to recover FAs from powdered ceramic and to determine their δ^13^C values. The protocol was then validated through analysis of archeological sherds with a variety of characteristics, and comparison of results with those achieved from contemporaneous analysis by established methods. The lipid profiles and δ^13^C FA values resulting from THM were analogous to those obtained from the acidified MeOH protocol, while the concentrations of lipids recovered were equivalent to those achieved from solvent extraction (DCM/MeOH).

This minimally invasive approach based on THM enables a significant reduction in sample size and processing time, thereby widening the application of ORA by, for example (a) enabling the study of intact or valuable objects previously excluded by the sample size required, (b) permitting lipid profiling of different areas of one sherd to gain knowledge on lipid distribution, and (c) serving as a rapid screening method, for instance ahead of compound-specific radiocarbon dating.^79^

## Supplementary Material



## Notes

S1 additional details on instrument methods; S2 bar charts, DIP-GC-QToF-MS TICs, and amounts of lipids recovered from standard solutions; S3 archeological lipid profiles and amounts recovered; S4 carbon isotope composition of archeological fatty acids (PDF)
