# Supplementary material for: Thermal Desorption Gas Chromatography–Mass Spectrometry Methods for Minimally Invasive Organic Residue Analysis of Archeological Potsherds
Source: Anal Chem. 2025 Nov 13;97(46):25760–70. doi: 10.1021/acs.analchem.5c05331 (PMC12658860; doi:10.1021/acs.analchem.5c05331)
Supplement: Supplementary file 1 [file ac5c05331_si_001.pdf]

SUPPORTING INFORMATION

THERMAL DESORPTION GAS CHROMATOGRAPHY MASS SPECTROMETRY METHODS FOR MINIMALLY INVASIVE ORGANIC RESIDUE ANALYSIS OF ARCHEOLOGICAL POTSHERDS

Eugenia Geddes da Filicaia*[1,2], Charlie A. Maule[1], Alexander Nixon[1], David A. Peggie[2], Ian D. Bull[1], Richard P. Evershed[1], Mélanie Roffet-Salque*[1]

[1]Organic Geochemistry Unit, School of Chemistry, University of Bristol, Bristol BS8 1TS, U.K.;
[2]Scientific department, The National Gallery, Trafalgar Square, London WC2N 5DN, U.K.

Corresponding authors: email e.geddesdafilicaia@bristol.ac.uk and melanie.salque@bristol.ac.uk

TABLE OF CONTENTS

S1 Instrument methods....S1

S2 Recovery of lipids from standard solutions....S3

S3 Archeological lipid profiles and amounts recovered....S9

S4 Carbon isotope composition of archeological Fatty acids....S18

S1 INSTRUMENT METHODS

DIP-GC-C-IRMS. Analysis was carried out on an Agilent Technologies 7890B gas chromatograph coupled to an Elementar IsoPrime PrecisION isotope ratio mass spectrometer *via* an Elementar IsoPrime GC5 combustion interface (Elementar Analysensysteme GmbH, Hanau, Germany). The CuO and NiO wires reactor was maintained at 950 °C. The GC was equipped with a non-polar fused silica capillary column (30 m × 0.25 mm i.d. × 0.25 µm film thickness) with a BPX5 stationary phase (5% phenyl polysilphenylene-siloxane, SGE). Solid or liquid samples were loaded onto a quartz tube in the TSP, introduced in the multi-mode inlet (MMI) in splitless mode. The initial temperature was 50 °C, set to rise to 350 °C at a rate of 900 °C min^{-1}. The inlet was held at this temperature for 3 min before cooling to 300 °C at a rate of 900 °C min^{-1}, where it was held for 50 min. The oven temperature began at 40 °C, was increased to 200 °C (at 10 °C min^{-1}) and then to 310 °C (at 6 °C min^{-1}) with a final hold time of 20 min. He was used as carrier gas at constant flow (2 mL min^{-1}). The mass spectrometer was equipped with three Faraday cups collecting for the masses *m/z* 44 ($^{12}C^{16}O_2$), 45 ($^{13}C^{16}O_2$ and $^{12}C^{17}O^{16}O$) and 46 ($^{12}C^{18}O^{16}O$), with a solvent delay of 12 min. Data acquisition and processing were undertaken using the Lyticos software (Elementar, version 5.0.4.118). Reference CO_2 and in-house quality control standard made of FAMEs ($C_{11:0}$, $C_{13:0}$, $C_{16:0}$, $C_{18:0}$, $C_{21:0}$ and $C_{23:0}$) of known $\delta^{13}C$ values were used to monitor instrument performance. The FAME standard was also used for correction of determined $\delta^{13}C$ values using a two-point normalisation. Correction of $\delta^{13}C$ values due to the addition of C atoms from derivatisation was undertaken using the mass balance equation. All analyses were performed in duplicate.

DIP-GC-QToF-MS. Analysis was carried out on an Agilent Technologies 7890B gas chromatograph coupled to an Agilent Technologies 7200 Accurate Mass QTOF mass spectrometer (Agilent Technologies, Santa Clara, CA, USA). The GC was equipped with a non-polar fused silica capillary column (30 m × 0.25 mm i.d. × 0.25 µm film thickness) with a BPX5 stationary phase (5% phenyl polysilphenylene-siloxane, SGE). Solid or liquid samples were loaded onto a quartz tube in the TSP and introduced into the multi-mode inlet (MMI) in splitless mode. The initial temperature was 50 °C, set to rise to 350 °C at a rate of 900 °C min^{-1}. The inlet was held at this temperature for 1 min before cooling to 250 °C at a rate of 25 °C min^{-1}, where it was held for 40 min. The oven temperature began at 40 °C, was increased to 200 °C (at 10 °C min^{-1}) and then to 310 °C (at 6 °C min^{-1}) with a final hold time of 20 min. He was used as carrier gas at constant flow (1.2 mL min^{-1}). The mass spectrometer was set to acquire in full scan mode (*m/z* 50–1050) with an acquisition rate of 5 spectra s^{-1} and an acquisition time of 200 ms spectrum^{-1}, and a solvent delay of 6.5 min. The emission current was 2.1 µA. The MS was operated at an EI potential of 70 eV for standard ionisation experiments. For MS/MS experiments, low energy ionisation was utilised, with N_2 as the collision gas with collision energy of 10 eV (or 20 for *m/z* lower than 80). In this case, the mass range was set to (*m/z* 50–300), the acquisition time to 300 ms spectrum^{-1}. The MS transfer line temperature was set to 320 °C, the ion source temperature to 230 °C, and the quadrupole to 150 °C. Data acquisition was undertaken using Agilent Technologies MassHunter GC/MS Acquisition (version B.07.02.1938), and data processing with Agilent Technologies MassHunter software (version 10.0). Peaks were identified using a combination of mass spectral information, GC retention times, and comparison to the NIST mass spectral library (Mass Spectral Search Program version 2.0 and NIST 08 MS Library), published datasets, and NG databases. An in-house quality control standard made of FAMEs ($C_{14:0}$, $C_{15:0}$, $C_{16:0}$, $C_{18:0}$ and $C_{20:0}$) and an *n*-alkane ladder standard, containing C_7–C_{40} saturated alkanes (0.001 mg mL^{-1} in *n*-hexane), was used to monitor instrument performance.

GC-C-IRMS. Analysis was carried out using the same method as for DIP-GC-C-IRMS, except for the absence of the TSP. Instead, extracts (1 µL) were injected into the multi-mode inlet (MMI) in splitless mode with an inlet temperature beginning at 50 °C and rising to 300 °C at a rate of 900 °C min^{-1} (held for 53 min).

GC-FID. Screening of FAME derivatised total lipid extracts (TLEs) was performed on either a Hewelett Packard 5890 series II gas chromatograph or an Agilent Technologies 7820A GC (Agilent Technologies, Santa Clara, CA, USA) equipped with a non-polar fused silica capillary column (50 m × 0.32 mm i.d. × 0.17 µm film thickness) with a HP-1 stationary phase (100% dimethylpolysiloxane, Agilent Technologies). Extracts (1 µL) were injected on-column at an oven temperature of 50 °C. After an isothermal hold for 2 min, the oven temperature was increased to 300 °C at a heating rate of 10 °C min^{-1}, then held for 15 min. He was used as carrier gas at constant flow (2 mL min^{-1}), and a flame ionisation detector (FID), set to 300 °C, used to monitor column effluent. Quantification was performed by the addition of an internal standard (IS), C_{34} *n*-alkane (20 µg), added for quality control during the sample preparation stage. Data acquisition and processing were conducted using Agilent MSD ChemStation software (F.01.01.2317, Agilent Technologies). An in-house quality control standard made of FAMEs ($C_{14:0}$, $C_{15:0}$, $C_{16:0}$, $C_{18:0}$ and $C_{20:0}$) was used to monitor instrument performance.

GC-MS. Derivatised TLEs were diluted in *n*-hexane (*ca.* 0.1 mg mL^{-1}) and analysed using a Thermo Scientific TRACE 1300 GC interfaced with an ISQ LT single quadrupole mass spectrometer (ThermoScientific, Hemel Hempstead, UK). The GC was equipped with a non-polar fused silica capillary column (50 m × 0.32 mm i.d. × 0.17 µm film thickness) with an HP-1 stationary phase (100% dimethylpolysiloxane, Agilent Technologies). Extracts (1 µL) were injected into a PTV injector in splitless mode, with an inlet temperature beginning at 50 °C and rising to 300 °C at a rate of 14.5 °C s^{-1} (held for 35 min). The oven temperature began at 50 °C (2 min isothermal hold) and was increased to 300 °C at a heating rate of 10 °C min^{-1}, then held for 15 min. He was used as carrier gas at constant flow (2 mL min^{-1}). The mass spectrometer was set to acquire in the full scan mode (*m/z* 50–650) at 0.2 scan s^{-1}, with a solvent delay of 8 min. The MS was operated at an electron ionisation (EI) potential of 70 eV. The MS transfer line and ion source temperatures were kept at 300 °C. Data acquisition and processing were undertaken using Xcalibur software (4.1.31.9, ThermoFisher Scientific). Peaks were identified using a combination of mass spectral information, GC retention times, and comparison to the NIST mass spectral library (Mass Spectral Search Program version 2.0 and NIST 08 MS Library), and published datasets. An in-house quality control standard made of FAMEs ($C_{11:0}$, $C_{13:0}$, $C_{16:0}$, $C_{18:0}$, $C_{21:0}$ and $C_{23:0}$) was used to monitor instrument performance.

GC-QToF-MS 'aquatic biomarker method'. Analysis was carried out on an Agilent Technologies 7890B gas chromatograph coupled to an Agilent Technologies 7200 Accurate Mass quadrupole-time of flight (QTOF) mass spectrometer (Agilent Technologies, Santa Clara, CA, USA). The GC was equipped with a non-polar fused silica capillary column (50 m × 0.32 mm i.d. × 0.17 µm film thickness) with an HP-1 stationary phase (100% dimethylpolysiloxane, Agilent Technologies). Extracts (1 µL) were injected in a multi-mode inlet (MMI) in splitless mode with an inlet temperature beginning at 70 °C and rising to 300 °C at a rate of 900 °C min^{-1} (held for 70 min). The oven temperature began at 50 °C, and, after an isothermal hold of 2 min, it was increased to 160 °C at a heating rate of 10 °C min^{-1}, then to 300 °C at a heating rate of 3 °C min^{-1}, where it was held for 5 min. He was used as carrier gas at constant flow (2 mL min^{-1}). The mass spectrometer was acquiring in full scan mode (*m/z* 50–1050) with an acquisition rate of 5 spectra s^{-1} and an acquisition time of 200 ms $spectrum^{-1}$, and a solvent delay of 5 min. The emission current was 2.1 µA. The MS was operated at an EI potential of 70 eV. The MS transfer line temperature was set to 320 °C, the ion source temperature to 230 °C, and the quadrupole to 150 °C. Data acquisition was undertaken using Agilent Technologies Chemstation, and data processing with Agilent Technologies MassHunter software (version 10.0). Peaks were identified using a combination of mass spectral information, GC retention times, and comparison to the NIST mass spectral library (Mass Spectral Search Program version 2.0 and NIST 08 MS Library) and published datasets. An in-house quality control standard made of FAMEs ($C_{14:0}$, $C_{15:0}$, $C_{16:0}$, $C_{18:0}$ and $C_{20:0}$) was used to monitor instrument performance.

HT-GC-FID. Screening of trimethylsilylated (TMS) derivatised TLEs was undertaken on an Agilent Technologies 7890A gas chromatograph (Agilent Technologies, Santa Clara, CA, USA) equipped with a non-polar fused silica capillary column (15 m × 0.32 mm i.d. × 0.1 µm film thickness) with a DB1-HT stationary phase (100% dimethylpolysiloxane, Agilent Technologies). Extracts (1 µL) were injected on-column at an oven temperature of 50 °C. After an isothermal hold for 2 min, the oven temperature was increased to 350 °C at a heating rate of 10 °C min^{-1}, then held for 10 min. He was used as carrier gas at constant flow (4 mL min^{-1}), and a flame ionisation detector (FID), set to 350 °C, used to monitor column effluent. Where quantification was performed, this was done through the addition of a C_{34} *n*-alkane IS (20 µg), added for quality control during the sample preparation stage. Data acquisition and processing were conducted using Agilent MSD ChemStation software (F.01.01.2317, Agilent Technologies). An in-house quality control standard made of trimethylsilylated glycolipids, containing a monoacylglycerol ($MAG_{16:0}$), a diacylglycerol ($DAG_{32:0}$), and three triacylglycerols ($TAG_{42:0}$, $TAG_{48:0}$, and $TAG_{54:0}$) was used to monitor instrument performance.

HT-GC-QToF-MS. Analysis was carried out on an Agilent Technologies 7890B gas chromatograph coupled to an Agilent Technologies 7200 Accurate Mass QTOF mass spectrometer (Agilent Technologies, Santa Clara, CA, USA). The GC was equipped with a non-polar fused silica capillary column (15 m × 0.25 mm i.d. × 0.1 µm film thickness) with a ZB-5HT Inferno stationary phase (5% phenyl polysilphenylene-siloxane, Phenomenex). Extracts (1 µL) were injected on-column at an oven temperature of 50 °C. After an isothermal hold for 2 min, the oven temperature was increased to 350 °C at a heating rate of 10 °C min^{-1}, then held for 10 min. He was used as carrier gas at constant flow (2.5 mL min^{-1}). The mass spectrometer was set to acquire in full scan mode (*m/z* 50–1050) with an acquisition rate of 5 spectra s^{-1} and an acquisition time of 200 ms $spectrum^{-1}$, and a solvent delay of 5 min. The emission current was 2.1 µA. The MS was operated at an EI potential of 70 eV. The MS transfer line temperature was set to 320 °C, the ion source temperature to 230 °C, and the quadrupole to 150 °C. Data acquisition was undertaken using Agilent Technologies MassHunter GC/MS Acquisition (version B.07.02.1938),, and data processing with Agilent Technologies MassHunter software (version 10.0). Peaks were identified using a combination of mass spectral information, GC retention times, and comparison to the NIST mass spectral library (Mass Spectral Search Program version 2.0 and NIST 08 MS Library) and published datasets. An in-house quality control standard made of FAMEs ($C_{14:0}$, $C_{15:0}$, $C_{16:0}$, $C_{18:0}$ and $C_{20:0}$) was used to monitor instrument performance.

S2 RECOVERY OF LIPIDS FROM STANDARD SOLUTIONS

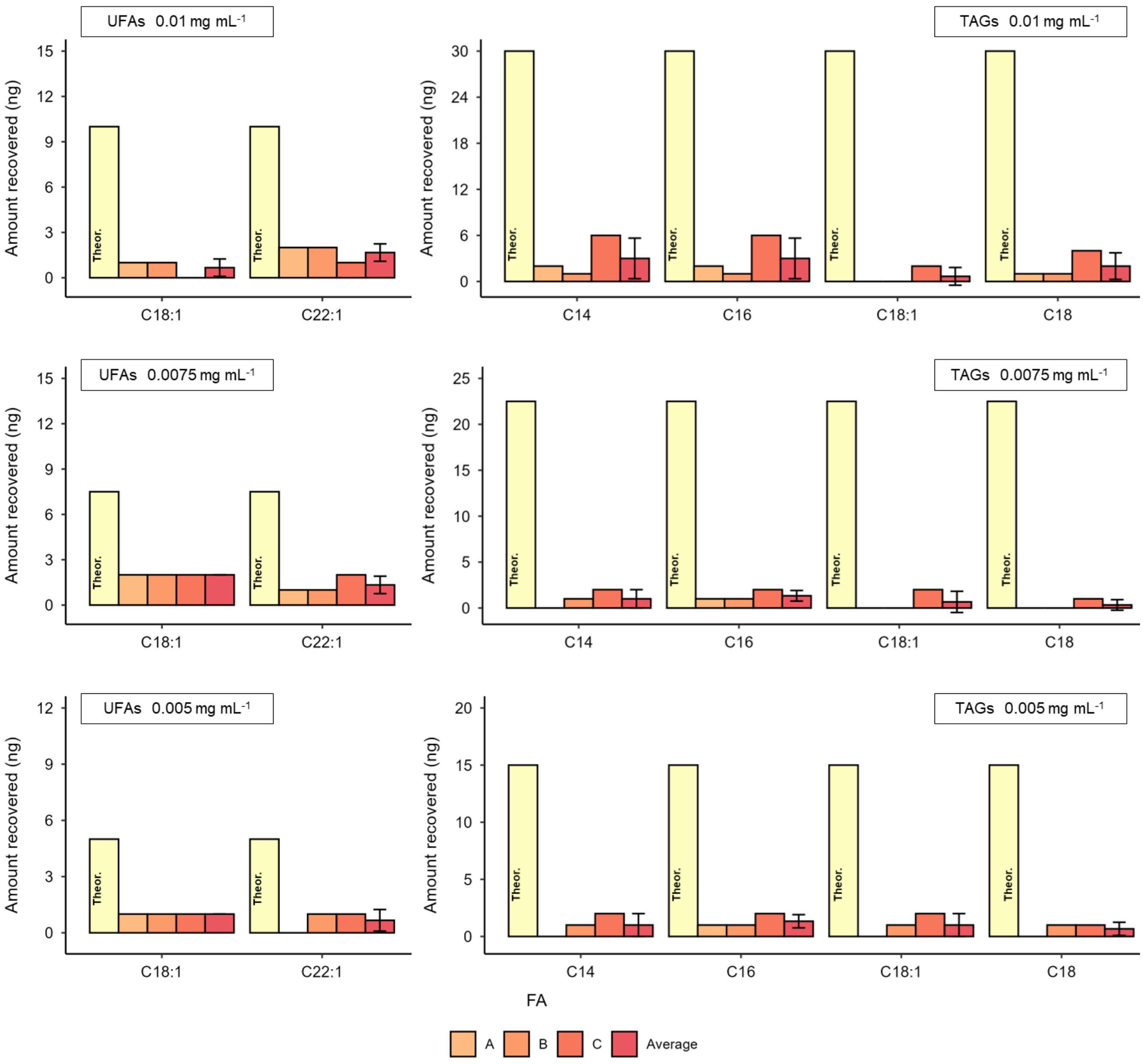

Figure S1. Amounts of FAs recovered from THM by DIP-GC-QToF-MS, performed in triplicate (A, B, C), of UFA and TAG standard solutions (1 μL). The average value is reported with the standard deviation (SD). The theoretical value (Theor.) is reported in yellow.

Table S1. Amounts of FAs recovered from THM by DIP-GC-QToF-MS, performed in triplicate (A, B, C), of FA standard solutions (1 μL). The average value is reported with the SD.

FAs standard solution		Amount of FAs in triplicate runs (A, B, C) of 1 μL each [ng]				
		Theoretical	Experimental			
			A	B	C	Average
0.005 mg mL^{-1}	$C_{14:0}$	5	5	4	4	4 (± 1)
	$C_{15:0}$	5	5	4	3	4 (± 1)
	$C_{16:0}$	5	6	4	3	5 (± 1)
	$C_{17:0}$	5	4	4	3	4 (± 1)
	$C_{18:0}$	5	4	4	3	4 (± 0)
	$C_{20:0}$	5	3	3	3	3 (± 0)
0.0075 mg mL^{-1}	$C_{14:0}$	7.5	5	6	15	9 (± 6)
	$C_{15:0}$	7.5	5	6	10	7 (± 3)
	$C_{16:0}$	7.5	5	6	11	7 (± 3)
	$C_{17:0}$	7.5	5	6	10	7 (± 2)
	$C_{18:0}$	7.5	5	6	10	7 (± 2)
	$C_{20:0}$	7.5	6	6	8	6 (± 1)
0.01 mg mL^{-1}	$C_{14:0}$	10	13	8	6	9 (± 3)
	$C_{15:0}$	10	12	8	6	9 (± 3)
	$C_{16:0}$	10	15	9	7	10 (± 4)
	$C_{17:0}$	10	12	9	7	9 (± 3)
	$C_{18:0}$	10	13	9	7	10 (± 3)
	$C_{20:0}$	10	12	10	8	10 (± 2)

Table S2: Amounts of FAs recovered from THM by DIP-GC-QToF-MS, performed in triplicate (A, B, C), of UFA standard solutions (1 μL). The average value is reported with the SD.

UFA standard solution		Amount of FAs in triplicate runs (A, B, C) of 1 μL each [ng]				
		Theoretical	Experimental			
			A	B	C	Average
0.005 mg mL^{-1}	$C_{18:1}$	5	1	1	1	1 (± 0)
	$C_{22:2}$	5	0	1	1	1 (± 0)
0.0075 mg mL^{-1}	$C_{18:1}$	7.5	2	2	2	2 (± 0)
	$C_{22:2}$	7.5	1	1	2	1 (± 0)
0.01 mg mL^{-1}	$C_{18:1}$	10	2	2	1	2 (± 1)
	$C_{22:2}$	10	2	2	1	1 (± 1)

Table S3. Amounts of FAs recovered from THM by DIP-GC-QToF-MS, performed in triplicate (A, B, C), of TAG standard solutions (1 μL). The average value is reported with the SD.

TAG standard solution		Amount of FAs in triplicate runs (A, B, C) of 1 μL each [ng]				
		Theoretical	Experimental			
			A	B	C	Average
0.005 mg mL^{-1}	$C_{14:0}$	5	0	1	2	1 (± 1)
	$C_{16:0}$	5	1	1	2	1 (± 1)
	$C_{18:1}$	5	0	1	2	1 (± 1)
	$C_{18:0}$	5	0	1	1	1 (± 0)
0.0075 mg mL^{-1}	$C_{14:0}$	7.5	0	1	2	1 (± 1)
	$C_{16:0}$	7.5	1	1	2	1 (± 1)
	$C_{18:1}$	7.5	0	0	2	1 (± 1)
	$C_{18:0}$	7.5	0	0	1	1 (± 0)
0.01 mg mL^{-1}	$C_{14:0}$	10	2	1	6	3 (± 2)
	$C_{16:0}$	10	2	1	6	3 (± 3)
	$C_{18:1}$	10	0	0	2	1 (± 1)
	$C_{18:0}$	10	1	1	4	2 (± 1)

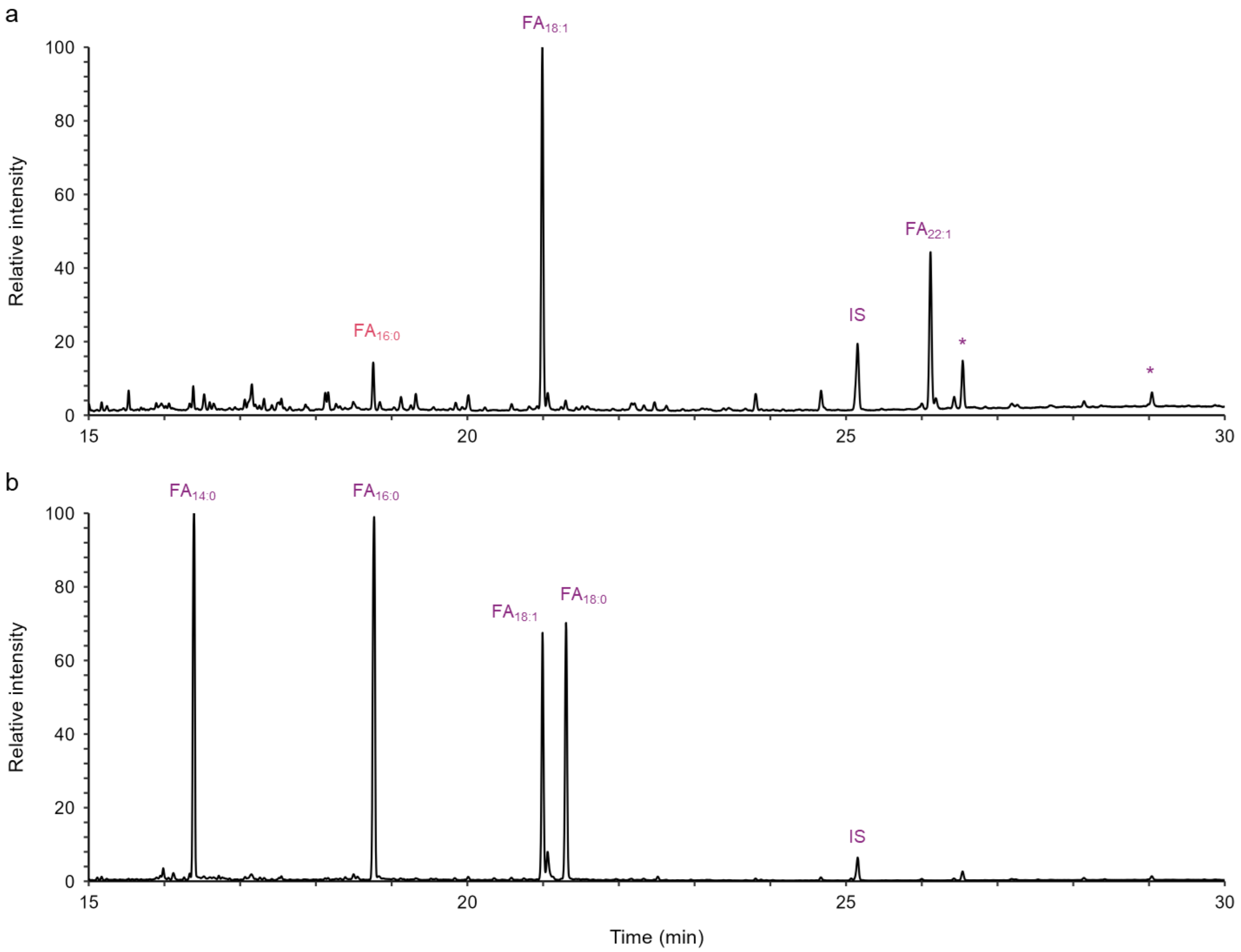

Figure S2. GC-MS partial TIC showing FAMEs recovered from THM by DIP-GC-QToF-MS of crushed modern sherd doped with (a) UFA, and (b) TAG standard solution (50 µg g^{-1}). FA = fatty acid (in pink when underivatised), IS = internal standard (henecosanoic acid), * = phthalate contamination.

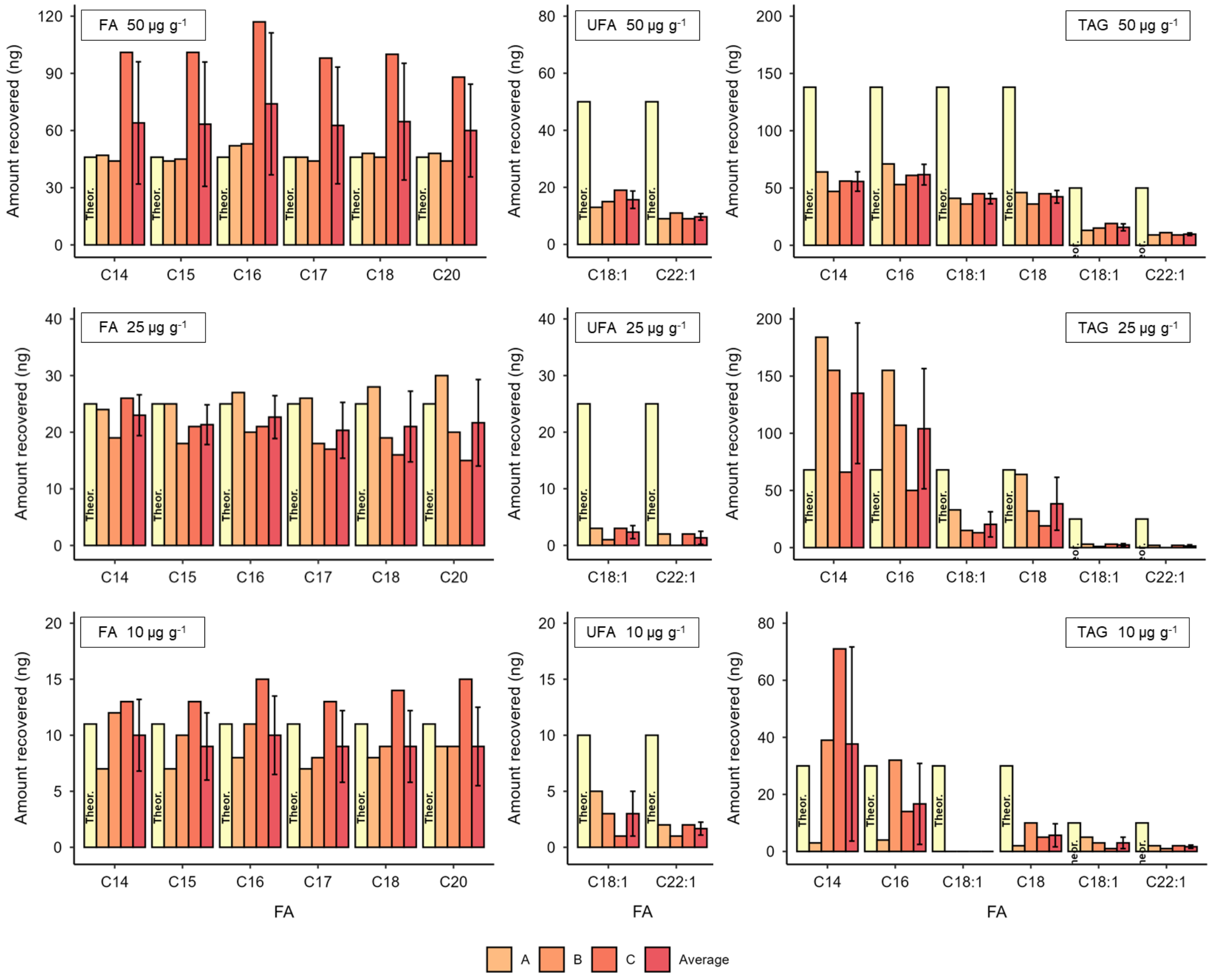

Figure S3. Amounts of FAs recovered from THM by DIP-GC-QToF-MS, performed in triplicate (A, B, C), of FA-, UFA-, and TAG-doped sherds (1 mg). The average value is reported with the SD. Theoretical (Theor.) amount expected in yellow.

Table S4. Amount of FAs recovered from THM with TMSH by DIP-GC-QToF-MS, performed in triplicate (A, B, C), of FA-doped sherd (1 mg), at 10, 25, and 50 µg g^{-1}. The average value is reported with the SD.

FAs doped sherd		Amount of FAs in triplicate runs (A, B, C) of 1 mg each [ng]				
		Theoretical	Experimental			
			A	B	C	Average
10 µg g^{-1}	$C_{14:0}$	11	7	12	13	10 (± 3)
	$C_{15:0}$	11	7	10	13	9 (± 3)
	$C_{16:0}$	11	8	11	15	10 (± 5)
	$C_{17:0}$	11	7	8	13	9 (± 3)
	$C_{18:0}$	11	8	9	14	9 (± 3)
	$C_{20:0}$	11	9	9	15	9 (± 4)
25 µg g^{-1}	$C_{14:0}$	25	24	19	26	23 (± 4)
	$C_{15:0}$	25	25	18	21	21 (± 4)
	$C_{16:0}$	25	27	20	21	22 (± 4)
	$C_{17:0}$	25	26	18	17	20 (± 5)
	$C_{18:0}$	25	28	19	16	21 (± 6)
	$C_{20:0}$	25	30	20	15	21 (± 8)
50 µg g^{-1}	$C_{14:0}$	46	47	44	101	64 (± 32)
	$C_{15:0}$	46	44	45	101	64 (± 33)
	$C_{16:0}$	46	52	53	117	74 (± 37)
	$C_{17:0}$	46	46	44	98	63 (± 31)
	$C_{18:0}$	46	48	46	100	65 (± 30)
	$C_{20:0}$	46	48	44	88	60 (± 24)

Table S5. Amount of FAs recovered from THM with TMSH by DIP-GC-QToF-MS, performed in triplicate (A, B, C), of UFA-doped sherd (1 mg), at 10, 25, and 50 µg g^{-1}. The average value is reported with the SD.

UFAs doped sherd		Amount of FAs in triplicate runs (A, B, C) of 1 mg each [ng]				
		Theoretical	Experimental			
			A	B	C	Average
10 µg g^{-1}	$C_{18:1}$	10	5	3	1	4 (± 2)
	$C_{22:1}$	10	2	1	2	2 (± 1)
25 µg g^{-1}	$C_{18:1}$	25	3	1	3	3 (± 1)
	$C_{22:1}$	25	2	0	2	1 (± 1)
50 µg g^{-1}	$C_{18:1}$	50	13	15	19	16 (± 3)
	$C_{22:1}$	50	9	11	9	10 (± 1)

Table S6. Amount of FAs recovered from THM with TMSH by DIP-GC-QToF-MS, performed in triplicate (A, B, C), of TAG-doped sherd (1 mg), at 10, 25, and 50 μg g^{-1}. The average value is reported with the SD.

TAGs doped sherd		Amount of FAs in triplicate runs (A, B, C) of 1 mg each [ng]				
		Theoretical	Experimental			
			A	B	C	Average
10 μg g^{-1}	$C_{14:0}$	30	3	39	71	38 (± 34)
	$C_{16:0}$	30	4	32	14	17 (± 14)
	$C_{18:1}$	30	0	0	0	0 (± 0)
	$C_{18:0}$	30	2	10	5	6 (± 4)
25 μg g^{-1}	$C_{14:0}$	68	184	155	66	135 (± 61)
	$C_{16:0}$	68	155	107	50	104 (± 53)
	$C_{18:1}$	68	33	15	13	20 (± 11)
	$C_{18:0}$	68	64	32	19	38 (± 23)
50 μg g^{-1}	$C_{14:0}$	138	64	47	56	56 (± 9)
	$C_{16:0}$	138	71	53	61	62 (± 9)
	$C_{18:1}$	138	41	36	45	40 (± 5)
	$C_{18:0}$	138	46	36	45	42 (± 6)

S3 ARCHEOLOGICAL LIPID PROFILES AND AMOUNTS RECOVERED

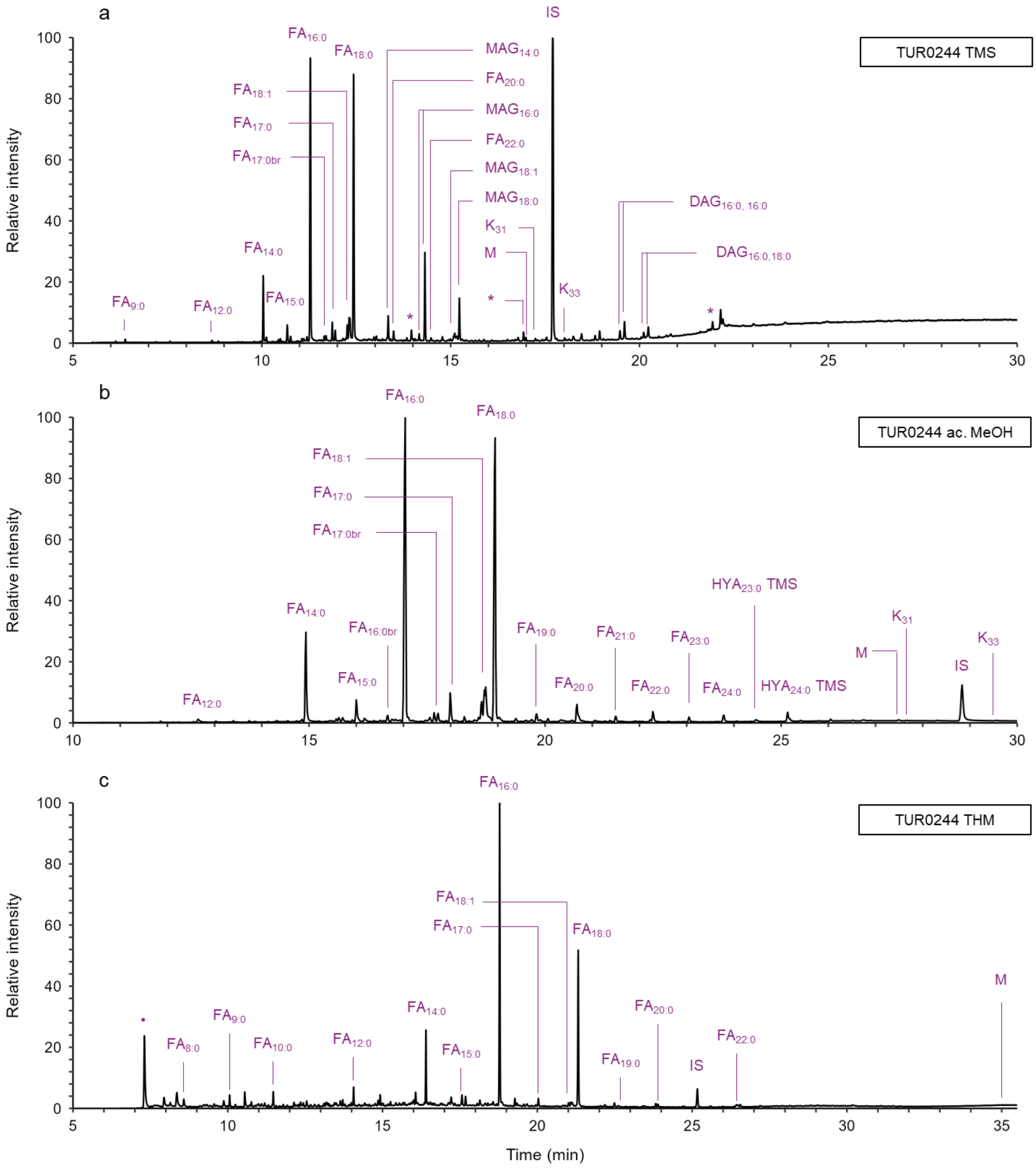

Figure S4. GC-MS partial TIC showing lipids recovered from sherd TUR0244, analyzed by solvent extraction (a), acidified MeOH (b), and THM (c). DAG = diacylglycerol, FA = fatty acid, IS = internal standard; tetratriacontane for (a) and (b), henecosanoic acid for (c), K = mid-chain ketone, HYA = hydroxy acid, M = miliacin, MAG = monoacylglycerol, • = TMSH degradation products, * = phthalate contamination.

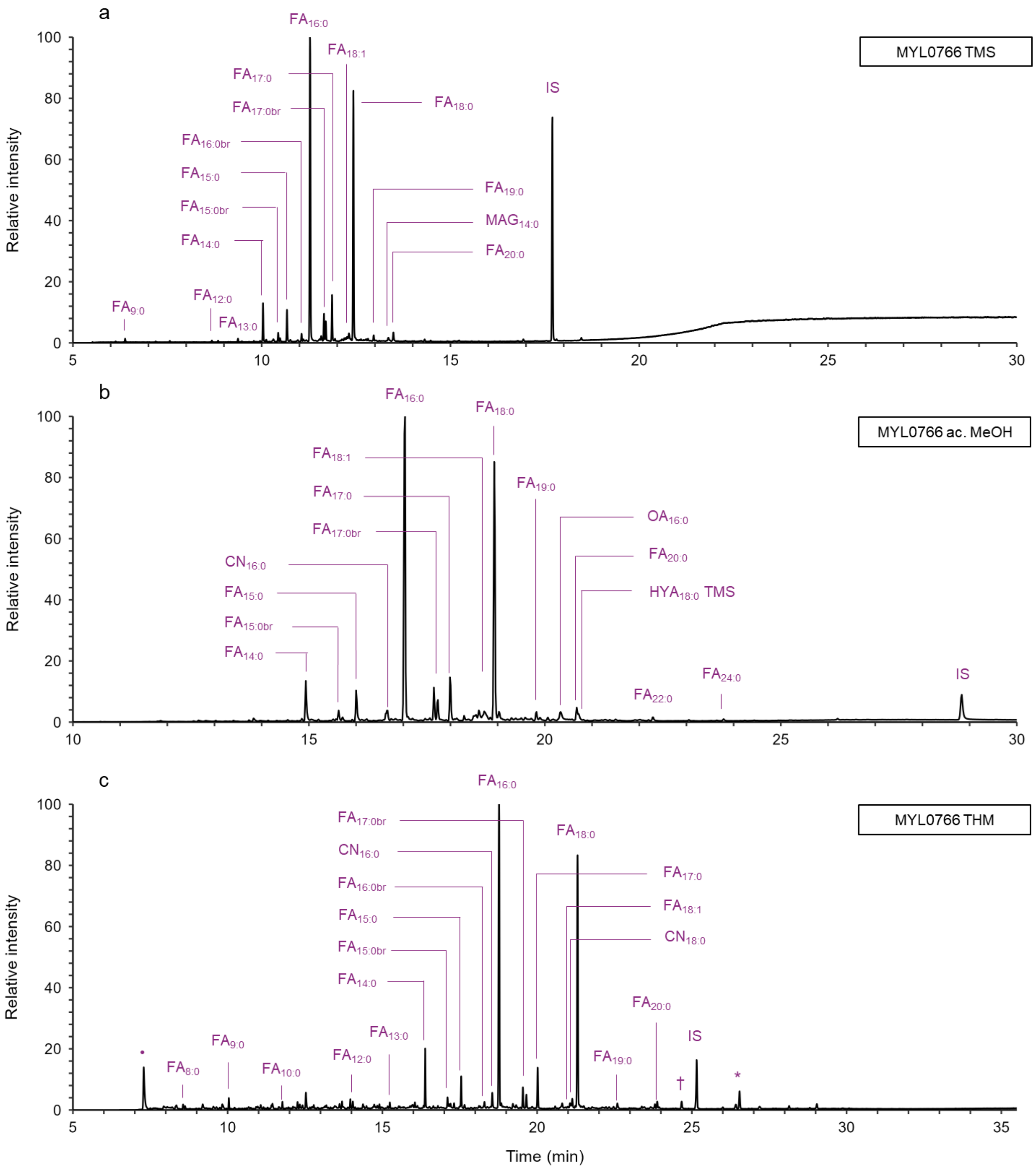

Figure S5. GC-MS partial TIC showing lipids recovered from sherd MYL0766, analyzed by solvent extraction (a), acidified MeOH (b), and THM (c). CN = aliphatic nitrile, FA = fatty acid, IS = internal standard; tetratriacontane for (a) and (b), henecosanoic acid for (c), HYA = hydroxy acid, MAG = monoacylglycerol, OA = ketone FA, • = TMSH degradation products, † = adipate contamination, * = phthalate contamination.

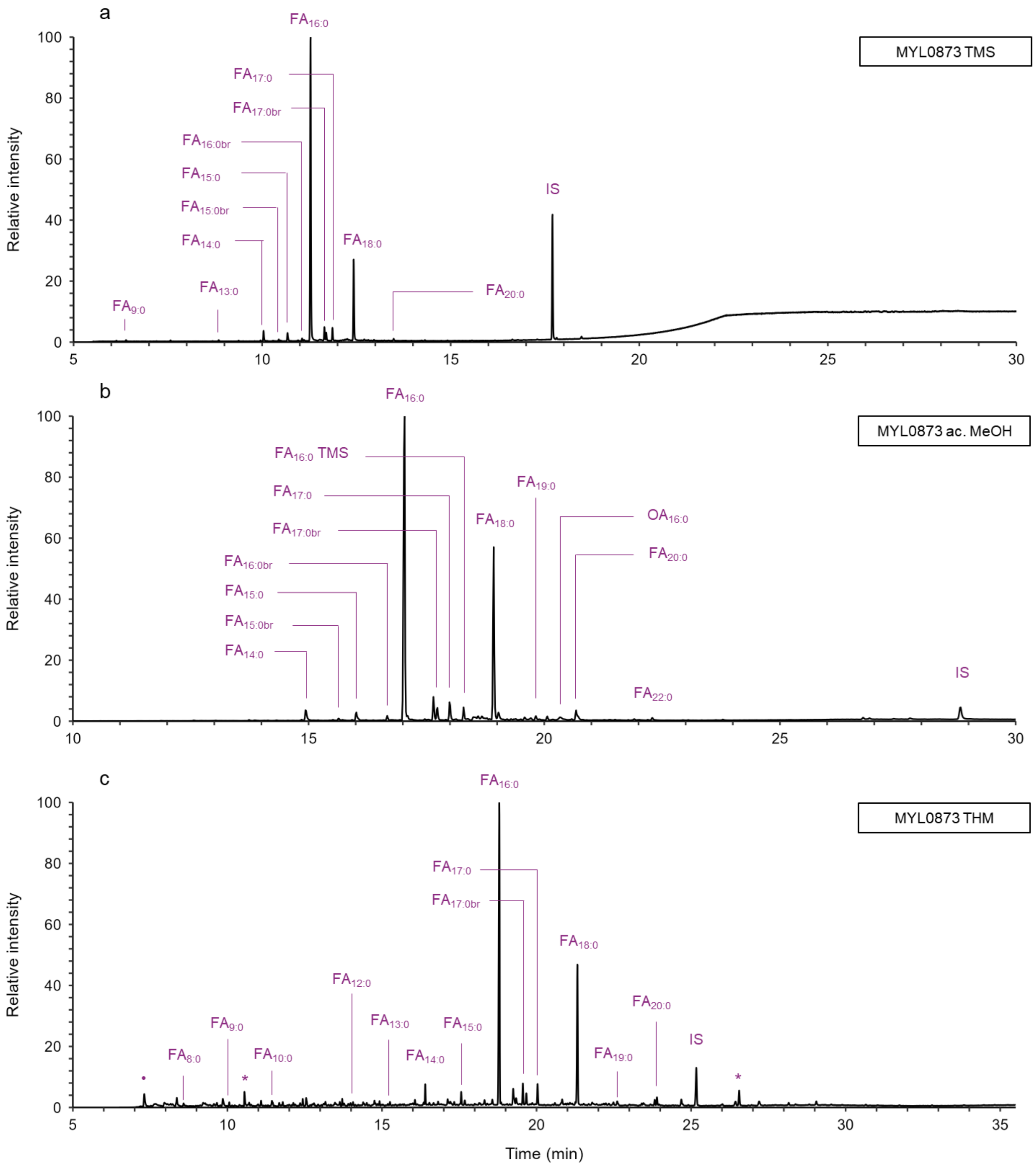

Figure S6. GC-MS partial TIC showing lipids recovered from sherd MYL0873, analyzed by solvent extraction (a), acidified MeOH (b), and THM (c). FA = fatty acid, IS = internal standard; tetratriacontane for (a) and (b), henecosanoic acid for (c), OA = ketone FA, • = TMSH degradation products, * = phthalate contamination.

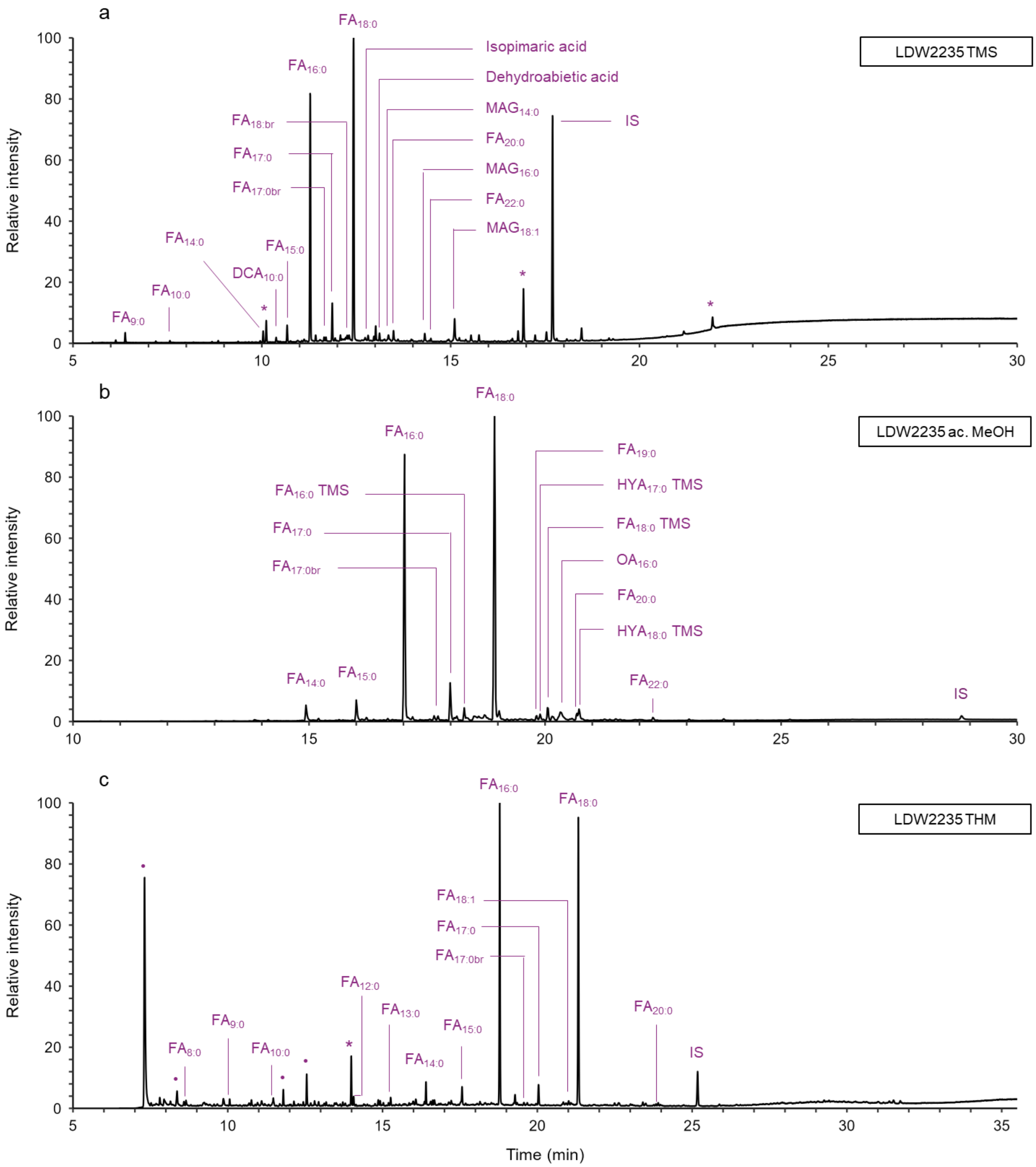

Figure S7. GC-MS partial TIC showing lipids recovered from sherd LDW2235, analyzed by solvent extraction (a), acidified MeOH (b), and THM (c). DCA = dicarboxylic acid, FA = fatty acid, IS = internal standard; tetratriacontane for (a) and (b), henecosanoic acid for (c), HYA = hydroxy acid, MAG = monoacylglycerol, OA = ketone FA, • = TMSH degradation products, * = phthalate contamination.

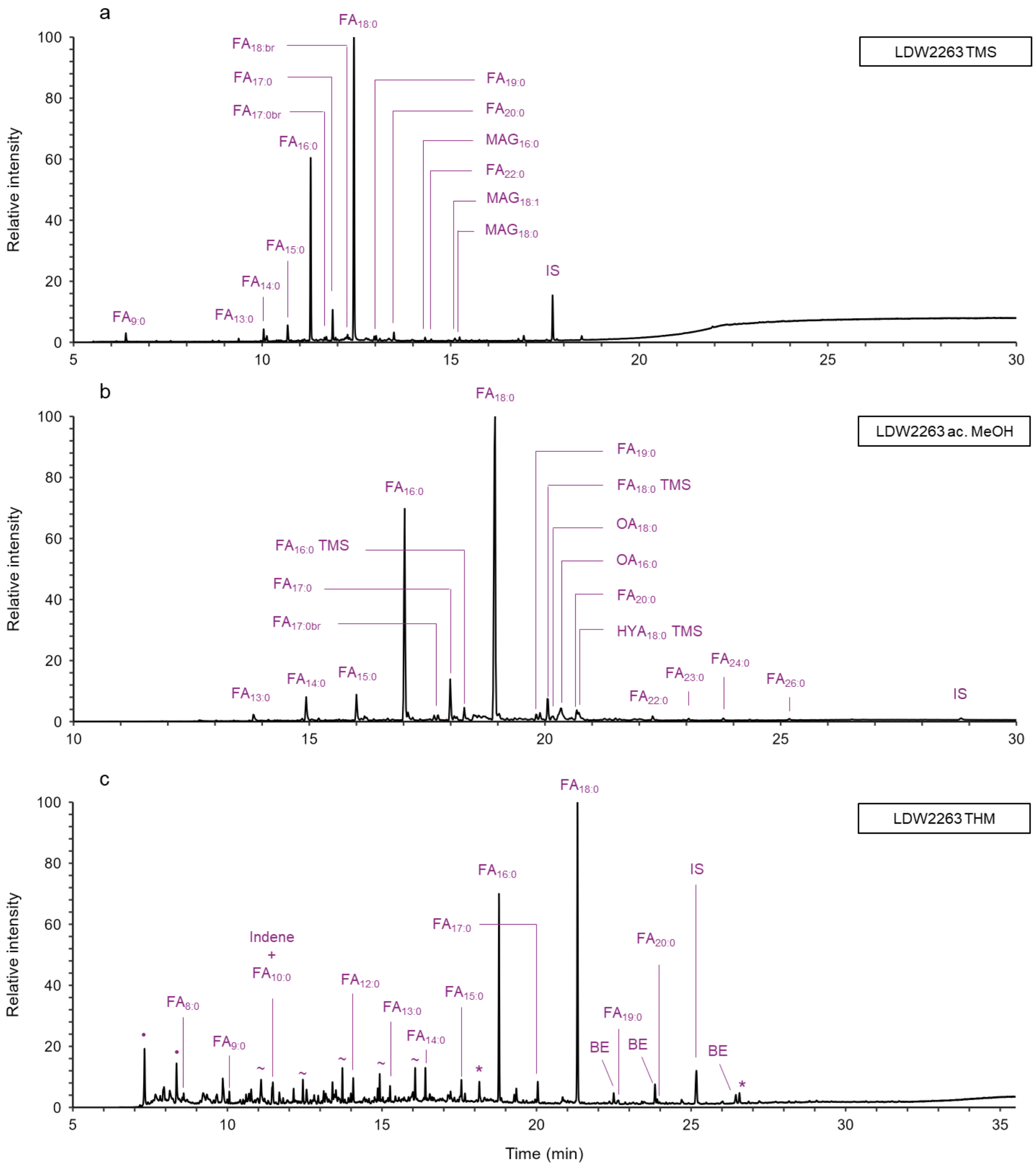

Figure S8. GC-MS partial TIC showing lipids recovered from sherd LDW2263, analyzed by solvent extraction (a), acidified MeOH (b), and THM (c). BE = benzoic acid long-chain ester, FA = fatty acid, IS = internal standard; tetratriacontane for (a) and (b), henecosanoic acid for (c), HYA = hydroxy acid, MAG = monoacylglycerol, OA = ketone FA, • = TMSH degradation products, ~ = alkane series, * = phthalate contamination.

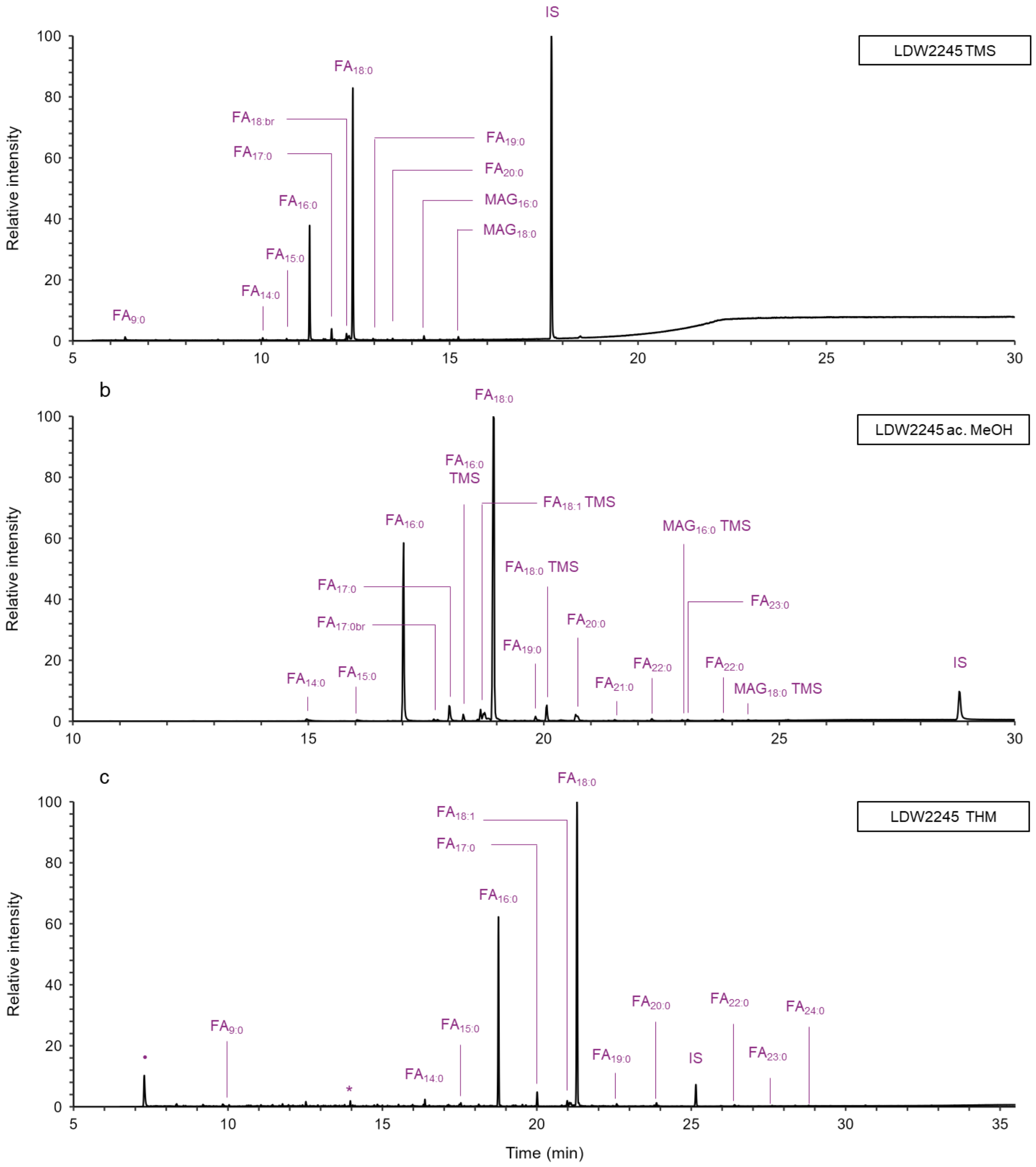

Figure S9. GC-MS partial TIC showing lipids recovered from sherd LDW2245, analyzed by solvent extraction (a), acidified MeOH (b), and THM (c). FA = fatty acid, IS = internal standard; tetratriacontane for (a) and (b), henecosanoic acid for (c), MAG = monoacylglycerol, • = TMSH degradation products, * = phthalate contamination.

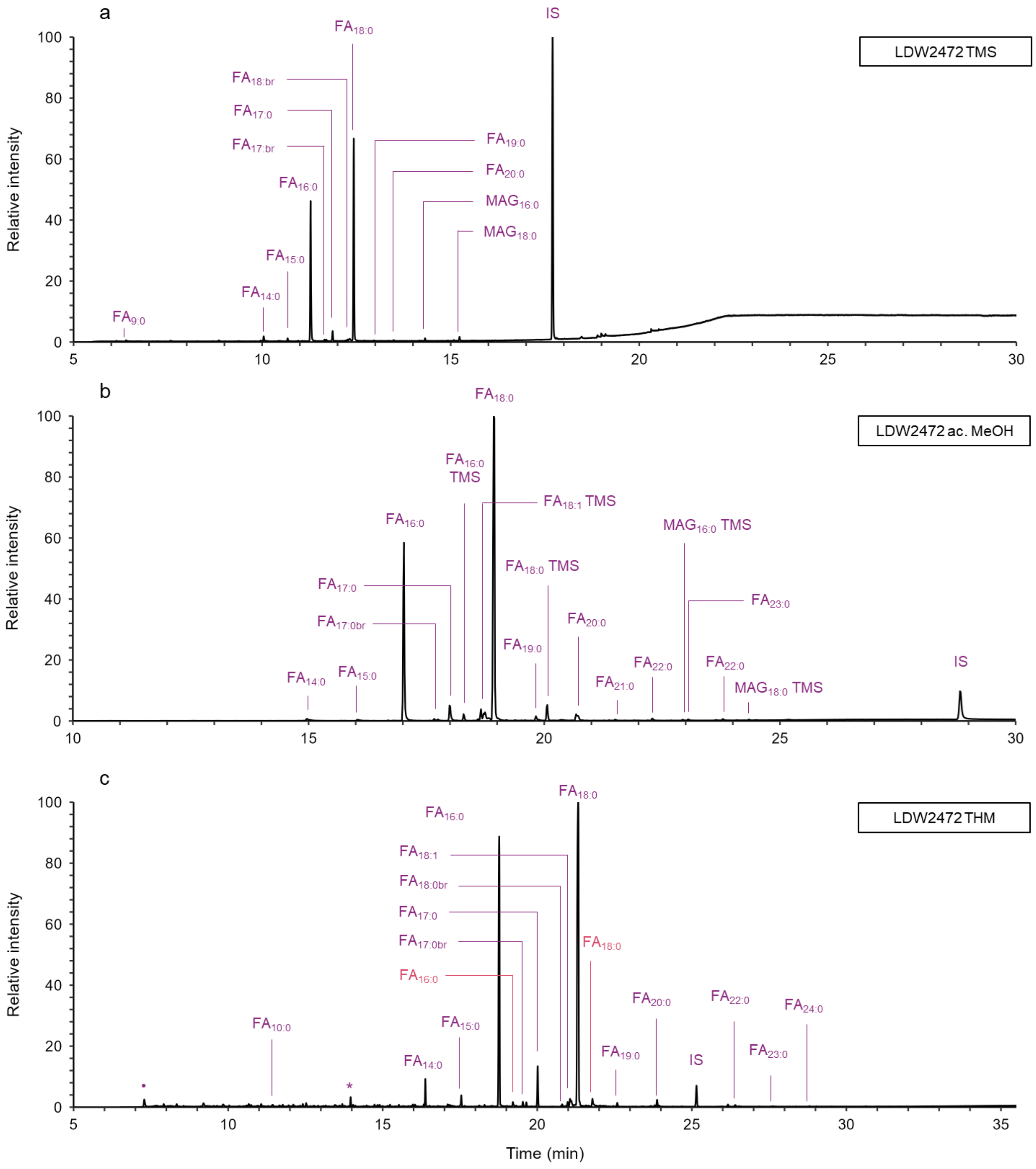

Figure S10. GC-MS partial TIC showing lipids recovered from sherd LDW2472, analyzed by solvent extraction (a), acidified MeOH (b), and THM (c). FA = fatty acid (in pink when underivatised), IS = internal standard; tetratriacontane for (a) and (b), henecosanoic acid for (c), MAG = monoacylglycerol, • = TMSH degradation products, * = phthalate contamination.

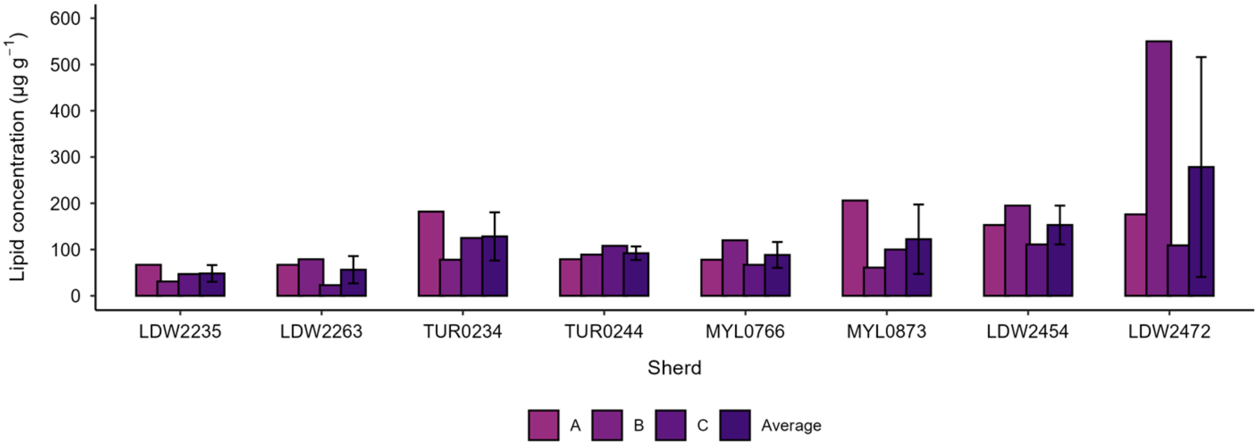

Figure S11. Lipid concentration for the archeological sherds, calculated from FAs recovered from THM by DIP-GC-QToF-MS, performed in triplicate (A, B, C). The average value is reported with the SD.

Table S7. Lipid concentration, calculated from lipids recovered from the archeological sherds, analyzed by solvent extraction, acidified MeOH, and THM. For the latter, amounts for each triplicate analysis (A, B and C) and the average value (with the SD) are given.

Potsherd	Concentration of lipids [µg g^{-1}]					
	THM with TMSH				DCM/MeOH (2:1 *v/v*)	MeOH (4% H_2SO_4 *v/v*)
	A	B	C	Average		
LDW2235	67	31	47	48 (± 18)	7	160
LDW2263	67	79	23	56 (± 29)	29	466
TUR0234	182	78	125	128 (± 52)	57	487
TUR0244	79	89	108	92 (± 15)	48	458
MYL0766	78	120	67	88 (± 28)	163	942
MYL0873	206	61	100	122 (± 75)	218	1226
LDW2454	153	195	111	153 (± 42)	117	1191
LDW2472	176	550	109	278 (± 238)	418	1583

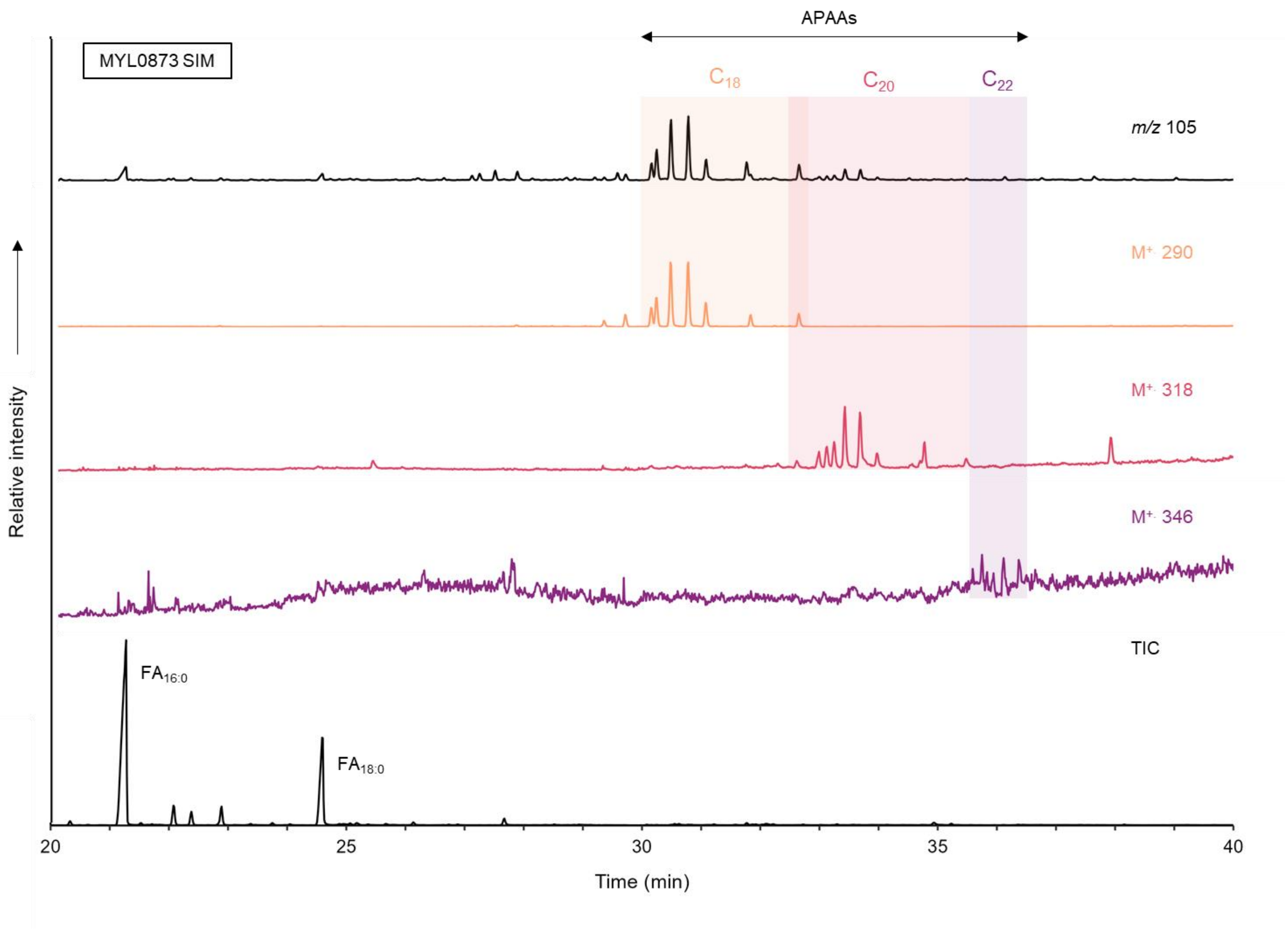

Figure S12: GC-MS partial TIC obtained from the extraction of sherd MYL0873 with acidified MeOH. The upper EICs obtained in SIM mode, correspond to the base peak (*m/z* 105, in black) and the molecular ions of APAAs C_{18} (M^+ 290, in orange), C_{20} (M^+ 318, in pink), and C_{22} (M^+ 346, in purple). FA = fatty acid.

Table S8. Molecular formulae, base peak (B^+) and molecular (M^+) ions for the three isoprenoid acids (IPAs) used as aquatic biomarkers.

IPA	Molecular formula	M^+	B^+	'aquatic biomarker method' RT (min)	THM RT (min)
TMTD	$C_{17}H_{34}O_2$	270.2559	87.0446	18.5	16.9
Pristanic acid ME	$C_{20}H_{40}O_2$	312.3029	88.0524	22.6	19.2
Phytanic acid ME	$C_{21}H_{42}O_2$	326.3185	101.0603	26.6	20.8

S4 CARBON ISOTOPE COMPOSITION OF ARCHEOLOGICAL FATTY ACIDS

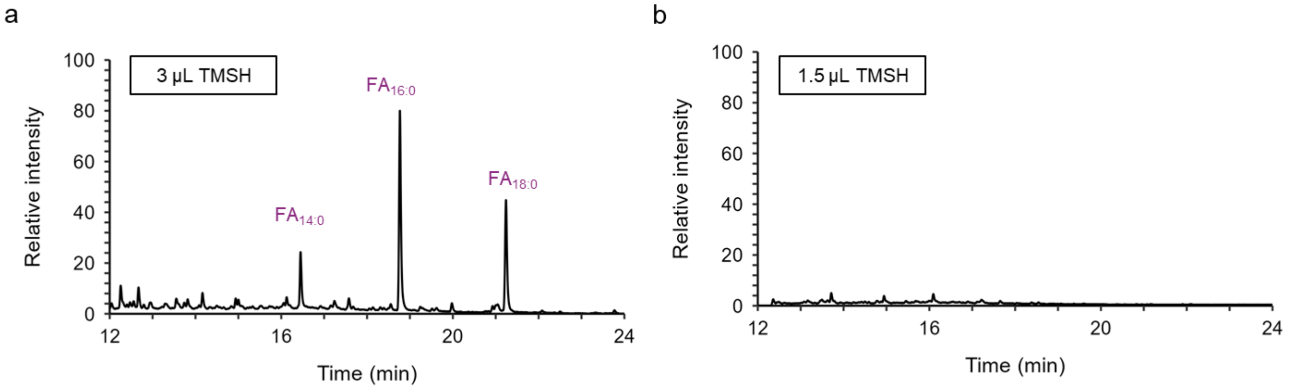

Figure S13. Partial TIC from THM by DIP-GC-C-IRMS of sherd TUR0244 (2 mg) with (a) 3 and (b) 1.5 µL of TMSH.

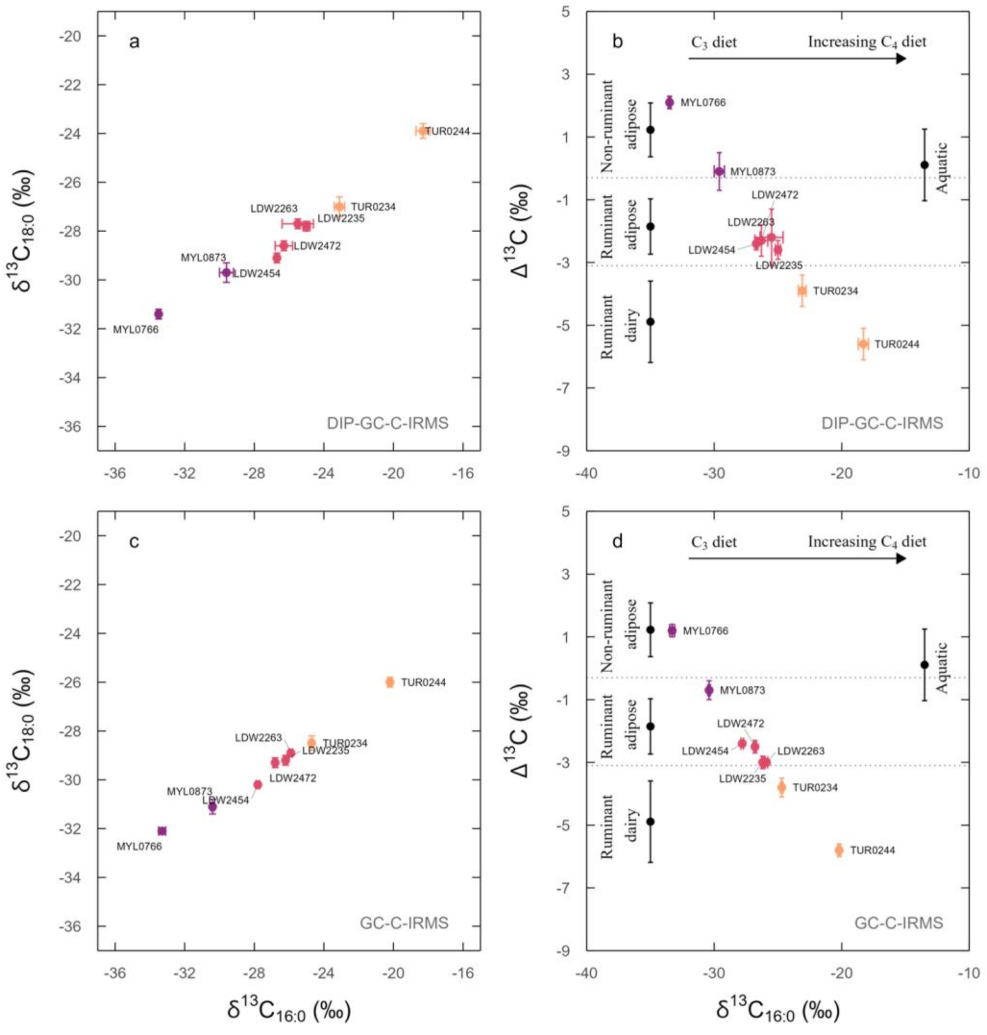

Figure S14. (a) Plot of the FA δ^{13}C values ($\delta^{13}C_{18:0}$ against $\delta^{13}C_{16:0}$) for the archeological sherds from Site 1 (pink), Site 2 (orange), and Site 3 (purple) obtained through the newly developed THM protocol (b) Plot of the corresponding Δ^{13}C values (Δ^{13}C against $\delta^{13}C_{16:0}$), showing the classification of animal fats as ruminant dairy for Site 2 (orange), ruminant adipose for Site 1 (pink) and non-ruminant adipose for Site 3 (purple). (c) and (d) are the identical plots, respectively, resulting from the δ^{13}C values obtained through the traditional acidified MeOH protocol. The error bars denote the SD on either side (±) of the value. Instrumental precision was 0.3‰

Table S9. $\delta^{13}C$ **values for** $C_{16:0}$ **and** $C_{18:0}$ **FAs obtained from the two protocols (THM with TMSH by DIP-GC-C-IRMS, and acidified MeOH by GC-C-IRMS), along the corresponding** $\Delta^{13}C$ **values for the eight archeological sherds investigated.**

Potsherd	GC-C-IRMS			TSP-GC-C-IRMS		
	$\delta^{13}C_{16:0}$ (‰)	$\delta^{13}C_{18:0}$ (‰)	$\Delta^{13}C$ (‰)	$\delta^{13}C_{16:0}$ (‰)	$\delta^{13}C_{18:0}$ (‰)	$\Delta^{13}C$ (‰)
TUR0234	−24.7 (± 0.1)	−28.5 (± 0.3)	−3.9 (± 0.3)	−23.1 (± 0.3)	−27.0 (± 0.4)	−3.9 (± 0.5)
TUR0244	−20.2 (± 0.1)	−26.0 (± 0.2)	−5.8 (± 0.2)	−18.3 (± 0.4)	−23.9 (± 0.3)	−5.6 (± 0.5)
MYL0766	−33.3 (± 0.2)	−32.1 (± 0.1)	1.2 (± 0.2)	−33.5 (± 0.1)	−31.4 (± 0.2)	2.1 (± 0.2)
MYL0873	−30.4 (± 0.1)	−31.1 (± 0.3)	−0.7 (± 0.3)	−29.6 (± 0.4)	−29.7 (± 0.4)	−0.1 (± 0.6)
LDW2235	−26.2 (± 0.1)	−29.2 (± 0.2)	−3.0 (± 0.2)	−25.0 (± 0.2)	−27.8 (± 0.2)	−2.6 (± 0.3)
LDW2263	−25.9 (± 0.1)	−28.9 (± 0.1)	−3.0 (± 0.1)	−25.5 (± 0.9)	−27.7 (± 0.2)	−2.2 (± 0.9)
LDW2454	−27.8 (± 0.1)	−30.2 (± 0.1)	−2.4 (± 0.1)	−26.7 (± 0.1)	−29.1 (± 0.2)	−2.4 (± 0.2)
LDW2472	−26.8 (± 0.1)	−29.3 (± 0.2)	−2.5 (± 0.2)	−26.3 (± 0.5)	−28.6 (± 0.2)	−2.3 (± 0.5)
